# Effect of Low Red‐to‐Far‐Red Light on Stem Elongation and Pith Cell Development in Dicots

**DOI:** 10.1002/pld3.70072

**Published:** 2025-04-15

**Authors:** Linge Li, Yorrit van de Kaa, Lotte van der Krabben, Ronald Pierik, Kaisa Kajala

**Affiliations:** ^1^ Experimental & Computational Plant Development Institute of Environmental Biology, Utrecht University Utrecht The Netherlands; ^2^ Current Affiliation: Dalian Yuanyi Technology Co., Ltd Dalian Liaoning China; ^3^ Current Affiliation: Laboratory of Molecular Biology Wageningen University & Research Wageningen The Netherlands

**Keywords:** central cylinder, elongation, pith, shade avoidance syndrome, stem, transcription factors

## Abstract

In dense canopies, light becomes a limiting factor for plant growth. Many plants respond to neighbor cues by growing taller to improve light capture, a phenomenon known as the shade avoidance syndrome (SAS). The major neighbor detection is via enrichment of far‐red (FR) light that leads to a low red:far‐red light ratio (R:FR), suppressing phytochrome activity. In tomato, low R:FR induces elongation of the internodes, but study into the role of different cell types in this response has remained limited.

We characterized changes in cellular anatomy of the tomato internode in response to low R:FR and its accompanying changes in gene expression. We observed changes to the pith traits, including increases in pith layer number, pith cell diameter, and longitudinal cell length. We profiled the transcriptome in the entire internodes and in the hand‐dissected pith in the central cylinder of the internode in response to low R:FR treatment and identified transcription factors (TFs) of interest that were upregulated in the central cylinder, mostly GATA, TCP, and bZIPs. We then characterized FR responses in eight dicotyledonous species. Significant pith elongation was observed in species that exhibited a strong internode elongation response. The FR‐responsive expression of homologs of target GATA, TCP, and bZIP TFs in the central cylinder was conserved within the Solanaceae family.

Overall, we discovered central cylinder gene expression patterns in SAS that are distinct from those of the entire internode, suggesting that some responses are unique and likely specific to vascular cell types such as pith. These patterns were conserved with close relatives of tomato but not in other dicot families we sampled, indicating that different molecular mechanisms drive FR responses in different dicots.

## 1 Introduction

The challenge of meeting the growing demands for food, fuel, and fiber in the face of increasing population and limited land area has led to the intensive cultivation of plants in dense vegetation. This approach, while maximizing the use of available land, introduces a significant problem: competition for limited resources, particularly light. Dense vegetation often triggers a phenomenon known as shade avoidance syndrome (SAS), where plants compete for light, leading to various morphological adaptations most commonly making the plant taller and more erect (Smith and Whitelam 1997). SAS is usually triggered by one of two common light cues: either by the enrichment of far‐red (FR) light in comparison to red (R) light as only FR is reflected from and passed through the neighboring or shading leaves or by depletion of blue light, indicating overall reduction of light intensity. Specifically, FR enrichment is detected through the ratio of R to FR light by phytochromes. Phytochromes are 120‐kD soluble proteins that have a covalently linked linear tetrapyrrole chromophore. They exist in two photointerconvertible forms, Pr and Pfr. Pr is the R light‐absorbing form, which can be converted to Pfr (the FR‐absorbing form) by the R light. Pfr is the active form of phytochrome and can be converted back to Pr by FR light (Reed et al. 1993; Kiss et al. 2003). The active form Pfr translocases from the cytosol into the nucleus and interacts with phytochrome‐interacting factors (PIFs) and facilitates their inactivation and degradation. PIFs are growth‐promoting transcription factors (TFs) involved in various physiological processes that accumulate in response to increase in FR light and lead to SAS phenotypes (Shin et al. 1997; Ruberti et al. 2012; Yang and Li 2017). The shade‐induced changes, including stem elongation and altered leaf architecture, have been observed across various plant species, such as *Arabidopsis*, *Brassica rapa* , and tomato (Osborne 1991; Pierik and De Wit 2014; Kohnen et al. 2016; Ballaré and Pierik 2017).

However, SAS is not the sole strategy for plant species to deal with low light. Exploring the strategies of plant species in different ecosystems reveals approaches contrasting to competing for light via SAS. Pioneer species and shade‐tolerant species demonstrate unique life strategies and can provide evolutionary perspectives on how plants respond to shade. These species exemplify the diverse ways plants thrive in various environments. Pioneer plants are equipped to establish themselves in harsh, sterile, and sun‐exposed environments, rapidly growing to utilize available resources before larger competitors can establish dominance (Miyazawa et al. 2014; Sottosanti 2023). However, as the environment changes and the intermediate species grow taller, the shade they cast can deprive the pioneer species of adequate sunlight (Sottosanti 2023). In response to shading, the composition of species transitions from pioneer plants to a mixed ecosystem. This ecosystem includes dominant plants that prefer high light and understory species that flourish in the shade provided by the forest canopy (Hagen et al. 2015; Avalos and Avalos 2019). How these different light‐use strategies, especially shade avoidance and tolerance, have evolved is not well characterized. SAS may have evolved alongside shade itself, potentially as early as the Late Devonian period, suggesting it could be more ancient than shade tolerance mechanisms in plants (Mathews 2006). In contrast, the evolution of shade tolerance is associated with attenuation of shade avoidance and reduced phenotypic plasticity in some dicot lineages such as milkweeds and *Geranium robertianum* (Gommers et al. 2017; Coverdale and Agrawal 2021). Therefore, there is evidence that in these lineages, SAS predates the evolution of shade tolerance. It would be reasonable to assume that SAS mechanisms are conserved between angiosperms, but this has not been extensively tested.

A lot of SAS research has been carried out in the model species *Arabidopsis thaliana* with a focus on cellular events in the epidermis as a driver of the petiole responses (de Wit et al. 2012; Pierik and De Wit 2014; van Gelderen et al. 2018). The *Arabidopsis* and its rosette growth habit however have their limitations, including not being a good model for stem elongation as a SAS trait. In this study, we set out instead to investigate how tomato ( *Solanum lycopersicum* ) and specifically its internodes respond to low R:FR. We paired organ‐level measurements with cellular anatomy, looking for the cell types that responded to low R:FR. We discovered changes in the pith cell length and layer number in response to low R:FR. We then profiled both the internode and the central cylinder (mainly composed of pith) transcriptomes to characterize the pith‐related transcriptional changes. Pith is a tissue located in the central cylinder of stems and composed of undifferentiated parenchyma cells (Zabel and Morrell 2020), currently hypothesized to have evolved through delayed and shortened protoxylem differentiation during early euphyllophyte evolution by Middle Devonian period (Tomescu and Mcqueen 2022). Hence, it is possible that pith evolution preceded that of SAS, so, here, we tested the hypothesis that pith plays a key role in internode elongation. Thus, we queried if pith morphological and transcriptomic responses to low R:FR were conserved within dicots of increasing distance to tomato. We assessed species from the Solanaceae, Brassicaceae, and Fabaceae and found some conservation in pith responses.

From the transcriptomics data, we identified three candidate TFs that responded to low R:FR in tomato internodes or in their pith. These were a GATA TF (*Solyc01g090760*), a TCP TF (*Solyc08g080150* or *SlTCP20*), and a bZIP TF (*Solyc07g053450*). We tested if their FR‐responsive behavior was conserved in our set of species and discovered Solanaceae‐specific conservation in these SAS responses. These family‐specific responses to low R:FR indicate that there is divergence between upregulated TFs and molecular mechanisms involved in stem elongation during SAS of different dicots. This highlights the need for diverse model species for understanding seemingly conserved plant responses to the environment.

## 2 Materials and Methods

### 2.1 Plant Materials and Growth Conditions

We germinated seeds of *S. lycopersicum* (cv Moneymaker [MM] [obtained from Intratuin B.V] and M82 [obtained from Tomato Genetics Resource Center]), *Capsicum annuum* , *Solanum melongena* (obtained from Intratuin B.V), *Pisum sativum* Cameor (obtained from Dr. Judith Burstin lab), and *Glycine max* (cv green shell) (obtained from https://www.dehobbytuinder.nl/) by placing them in sealed plastic containers lined with paper towels soaked in tap water for 1 week. After this germination period, we transplanted seedlings of uniform size into 7‐cm square pots filled with sieved Primasta soil. Approximately 1 week following the transfer, we randomly allocated the plants into two distinct groups and subjected them to either white light (WL) or supplemental far‐red light (WL + FR) treatments as described in Li et al. (2024). The seedlings were cultivated at a temperature of 20°C, maintaining a humidity level of 70%, under a 16‐h light/8‐h dark cycle. All plants were initially grown under standard WL conditions (with a balanced R:FR ratio) until they reached a uniform developmental stage. This ensured that all plants had similar physiological starting points before the FR light treatment (which was applied during the light period (16 h/day) and lasted for a week for the phenotypic measurements). We maintained the photosynthetically active radiation (PAR) value at 200 μmol/m^2^/s for both WL and WL + FR. For the WL + FR, we added FR light to the same PAR background to achieve a specific R:FR ratio of 0.2. The R:FR ratio was calculated to quantify the relative abundance of red light (660 nm) to FR light (730 nm) in the light spectrum. Spectral irradiance data were collected using a calibrated spectroradiometer or equivalent instrument. The red irradiance value was divided by the FR irradiance value to obtain the ratio. This method is consistent across all experiments involving all species, allowing for direct comparisons between species.

We sowed *A. thaliana* (Columbia‐0 [Col‐0]) and *Brassica nigra* seeds (Pantazopoulou et al. 2017) directly into the soil, treated them with a stratification period of 3 days in darkness at 4°C, and subsequently transferred them to the following conditions: LD: 16 h of light, 8 h of darkness, SD: 8 h of light, 16 h of darkness PAR of 150 μmol·m^−2^·s^−1^, an R/FR ratio of 2.0, a temperature of 20°C, and a relative humidity of 70%. Following a 7‐day growth period, we transplanted the seedlings into 70‐mL pots containing Primasta soil. At the age of approximately 2 months, we used Col‐0 plants in inflorescence stem elongation experiments, commencing when the inflorescence reached approximately 0.5 to 2 cm in length.

All experimental procedures were initiated at ZT = 3.5 h.

### 2.2 Phenotyping

We used a digital caliper to measure specific plant attributes accurately. For each species, we had eight replicates, and the experiments were conducted twice for consistency. In the case of *Arabidopsis*, the measurements were carried out with 12 replicates, and the experiment was repeated twice. The key phenotype measurements, specifically stem and internode length, were recorded every day at two fixed times, ZT = 3.5 h and ZT = 9.5.

### 2.3 RNA‐Seq

We harvested the first internode, located between the cotyledons and the first true leaf. We gathered groups of these internodes at intervals of 6, 24, 30, and 48 h following the commencement of the treatment at ZT = 3.5 h. Ultimately, this resulted in the collection of samples encompassing two lighting conditions (WL and WL + FR), two different cultivars (M82 and MM), four timepoints, and two types of tissues (internode and its central cylinder), each with a minimum of four biological replicates. Each biological replicate contained six to eight individual internodes or central cylinders of the internode. To preserve their integrity for mRNA isolation, the samples were promptly frozen in liquid nitrogen. RNA isolation, library construction, and high‐throughput sequencing were carried out exactly as we described in Li et al. (2024). Similarly, the analysis of the RNA‐seq (mapping and differential expression analysis) was conducted as we described in Li et al. (2024). Raw counts are available on NCBI GEO (GSE255611), and DEGs (adjusted *p*‐value < 0.05) and enriched GO terms are listed in Tables S1–S2. In our RNA‐seq analysis, we compared the M82 and MM tomato cultivars to identify intrinsic differences between these varieties (Figure S1). Our results did not reveal significant variations, with the exception of minor discrepancies observed in the central cylinder data. Therefore, for other analyses, we combined these two cultivars and use it as one species to further investigate species differences.

### 2.4 Histology

**FIGURE 1:**
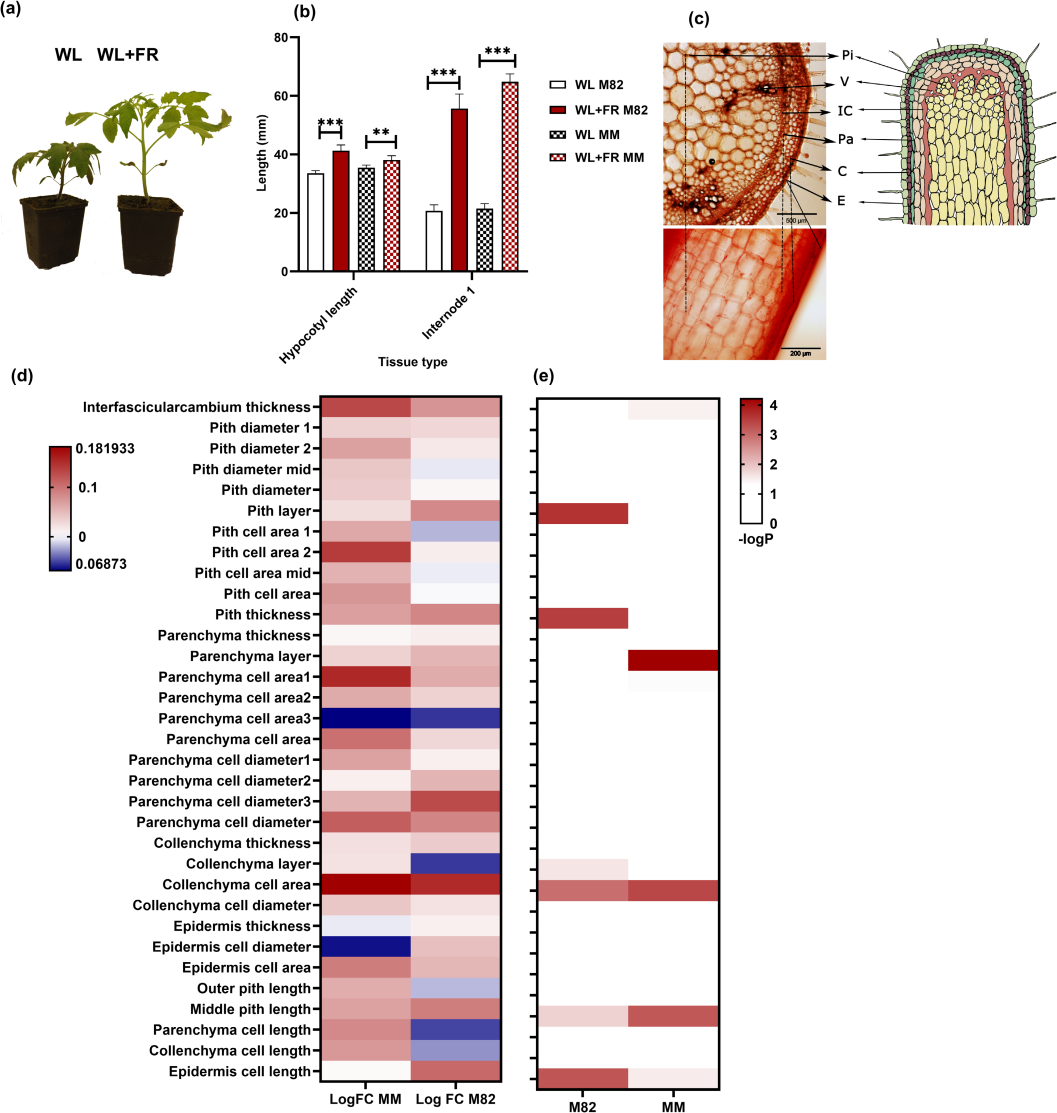
Tomato internode cellular morphology in response to low R:FR. (a) Comparison of *Solanum lycopersicum* cultivars under different light conditions. Two 24‐day‐old M82 plants after 10 days of treatment of either white light (WL) or white light supplemented with far‐red (WL + FR). (b) Stem length analysis in M82 and Moneymaker (MM) of 3‐week‐old plants following 7‐day treatments under WL and WL + FR. Notable differences between WL and FR + WL conditions are indicated with asterisks (* *p* ≤ 0.05; ** *p* ≤ 0.01; *** *p* ≤ 0.001). This analysis was conducted with 18 biological replicates and repeated three times. MM data are from Li et al. (2024). (c) Cellular anatomy of M82 first internode. The cross section details the cell types of interest including epidermis (E), collenchyma (C), parenchyma (Pa), and interfascicular cambium (IC), Vasculature (V, combined xylem and phloem) and pith (Pi). (d,e) FR‐responsive changes in cellular anatomy traits in the tomato first internode. The heatmaps show (d) logFC and (e) ‐log(*p*‐value) for each trait comparing WL + FR to WL. The layer number of each cell type is indicated from the outermost layer moving inward. Measurements of interfascicular cambium thickness were done in areas without vascular bundles. Cell length was captured from longitudinal sections, whereas the rest of the measurements were taken from cross sections. A logFC (logarithm of fold change) of 0 is depicted in white to signify no change, with upregulation shown in red and downregulation in blue. A‐log Pval of ‐log0.05 is set to white, indicating no statistical significance.

We stained the sections form the middle 1 cm of the first internode. We immersed the internode section into 0.1% (w/v) safranin solution was 2 min. Following this, we rinsed the samples with 70% ethanol repeatedly until the ethanol appeared clear. For long‐term preservation, we placed the samples back into 70% ethanol solution. We prepared sections of internode tissue with a vibratome (LEICA VT1000S) and imaged the sections with the Leica 700 Microscope with analyzeD software. We then measured specific parameters with ImageJ software as listed in Figure 1. All images were taken at a magnification of 10X. We used GraphPad Prism 9 tor the analysis and presentation of data. Furthermore, statistical analyses, including the use of the Student's *t*‐test, were performed with Microsoft Excel.

### 2.5 Promoter Cloning and Recombination

Promoter sequences were synthesized by GenScript (Table S3). Upon synthesis, we cloned these promoters into pENTR‐D/TOPO vectors (Invitrogen) through a topoisomerase I‐mediated cloning process and subsequently introduced into PMR074 destination vectors (Ron et al. 2014). This recombination was performed using the LR Clonase II Enzyme mix (Invitrogen), following the manufacturer's protocol for LR recombination. Each cloning and recombination step was verified by colony PCR and subsequent sequencing to confirm the integrity and correct orientation of the inserted promoter sequences. Final binary vector for plant transformation was then transformed into *Agrobacterium tumefaciens* AGL1 cells following protocol by Liu et al. (2023a), using Rif20/Strep50 selection and a colony PCR for verification.

### 2.6 Tomato Transformation

We sterilized the seeds of *S. lycopersicum* MM by immersing them briefly in 70% ethanol followed by a 20‐min dip in a 2.5% sodium hypochlorite solution. After multiple rinses with deionized water, we placed the seeds on a hormone‐free germination medium (GM) within sterile conditions and germinated them under long‐day conditions at 20°C and 70% relative humidity.

The GM we used was composed of 0.49% Murashige and Skoog (MS) basal medium supplemented with vitamins, 2‐morpholinoethanesulfonic acid (MES), 2% sucrose, and 0.8% plant agar, pH 5.8. We then used cotyledons and the first real leaves from sterile tomato seedlings aged 1–2 weeks as explant material for transformation. A single colony of *A. tumefaciens* was cultured in LB medium supplemented with appropriate antibiotics overnight at 28°C. We resuspended the culture in inoculation buffer MMA (0.49% MS with vitamins + MES, 100‐μM acetosyringone at pH 5.5–5.7) to an OD600 of 0.5 and inoculated the plant material with this for 20 min. We then co‐cultivated the explants on GM infused with antibiotics at 22°C in the dark for 2 days. Antibiotics we used for selection included 20‐mg/L Hygromycin, 250‐mg/L Cefotaxime, and 150‐mg/L Timentin.

For callus/shoot regeneration from cotyledons, we followed the methodology described by McCormick (1991), Sun et al. (2006), Gupta and Van Eck (2016) andSandhya et al. (2022). First, we selected for positive transformants on callus medium (CM; 1xMS with vitamins, MES, 3% sucrose, 1.5‐mg/L zeatin, 0.1‐mg/L IAA, and 0.8% plant agar, pH 5.8) containing suitable antibiotics in the dark at 21zipper‐like–26°C. Then, we transferred the developed callus with adventitious buds to shoot elongation medium (SM) in light, which mirrored the CM in composition with the addition of 0.5–1‐mg/L zeatin. Then we transferred the developed shoots to rooting medium (RM) under long‐day photoperiod to promote rooting.

### 2.7 GUS Staining Assay

We characterized the promoter activity patterns with the first‐generation transformed (T1) seeds. We grew the T1 plants 2 weeks in long‐day conditions and subsequently treated them with WL and WL + FR for 6 h (ZT = 3 to 9 h). We assessed the activity of the β‐glucuronidase (GUS) gene with a histochemical GUS staining assay. We carried out the staining with fresh staining solution (50‐mM NaPO4, 4‐mM potassium ferricyanide (III), 4‐mM potassium ferrocyanide (II), 0.05% (v/v) Triton X‐100, 0.05% (w/v) X‐Gluc). We submerged the seedlings in the freshly prepared staining solution and applied vacuum infiltration for 10–15 min to ensure thorough penetration. Following infiltration, the samples were incubated in darkness at 37°C for 24 h when blue color started to appear. Plant tissue was washed with MQ water to remove excess stain, followed by a series of ethanol washes (50%, 70%, and 96%) for clearing, which could be extended to an overnight duration if necessary. We then visualized the plants with microscopy and prepared sections as described above.

### 2.8 Quantitative RT‐PCR

To quantify changes to the transcript levels in response to supplemental FR, we used quantitative Real‐Time (qRT) PCR. We subjected the plants to two different lighting conditions: exposure to WL and a low R/FR ratio of 0.2 (WL + FR). Post 6 or 24 h of light treatment, we harvested the plant material, focusing on either the whole internode or the central cylinder hand dissected from the internode. We replicated this experiment four times, and for qPCR, we had three technical replicates. For each biological replicate, three internodes were collected into a tube, rapidly frozen in liquid nitrogen, and then stored at −80°C. We extracted total RNA using the RNeasy kit (Qiagen) and synthesized cDNA with random hexamer primers and RevertAid H Minus Reverse Transcriptase. We carried out real‐time PCR with primers detailed in Table S4 and SYBR Green Super mix (Thermo Fisher Scientific) on the Bio‐Rad CFX Opus 384 system. To quantify the relative transcript abundance, we applied the comparative 2^ΔΔCt^ method, using *ACTIN2* as the reference gene for normalization (Reynoso et al. 2019). To statistically analyze the qPCR data, we carried out ANOVA followed by a Tukey post hoc test using R software. For data presentation and visualization, we used GraphPad software (Version 9.5.1, released on January 24, 2023).

### 2.9 Homolog Analysis

In our study, we conducted a comprehensive homolog search across various plant species belonging to different families. We used the default BLAST search settings on several databases to source cDNA sequences. For *Arabidopsis*, we obtained sequences from TAIR (The Arabidopsis Information Resource 2015). Soybean ( *G. max* ) and *P. sativum* sequences we sourced from Phytozome (Goodstein et al. 2012). Solanaceae family sequences we accessed from Sol Genomics (Solgenomics.net) (Mueller et al. 2005), which included ITAG4.0 for *S. lycopersicum* , *C. annuum* UCD 10X genome chromosome v1.0 for *C. annuum* , and eggplant V4 for *S. melongena* . Additionally, orthologs for *B. nigra* we obtained from the Brassica Database (http://brassicadb.cn/#/BLAST/).

In our methodology for identifying orthologs, we prioritized the highest score of similarity, maintaining a threshold of over 70%. In cases where the similarity difference was less than 10%, we included multiple homologs.

### 2.10 Phylogenetic Analyses

For our study, we aligned the homologs of all TFs using Muscle v3.8.31 (Edgar 2004). Following this alignment, we generated phylogenetic trees using the Maximum Likelihood method in the Mega X software (Kumar et al. 2016). This process was based on the Tamura‐Nei model for constructing the ML tree. To ensure the robustness of our phylogenetic analysis, we performed a bootstrap analysis with 1000 rapid bootstraps. Subsequently, we applied the consensus tree method to select representative genes for each species. We used the resulting output file from this process to construct a consensus tree following a majority rule consensus approach. This step was crucial to ensure that only nodes with bootstrap support values exceeding 50% were included in our final phylogenetic tree. We carried out the visual representation and fine‐tuning of the ML analysis outcomes using Figtree v1.4.3 (Rambaut 2012).

## 3 Results

### 3.1 Stem and Pith Cells Elongated in Response to FR in Tomato

We characterized the tomato internode responses to low R:FR. To ensure our findings would be representative of tomato cultivars, we included tomatoes specifically bred for two different cultivation systems: M82 as a model for processing tomatoes and MM as a greenhouse model. Our objective was to conduct detailed phenotyping on young plants to explore various aspects of stem responses at both the macroscopic and cellular levels. After transplanting germinated seedlings into soil and allowing a week for recovery, we subjected them to 7‐day light treatments, either WL or white light supplemented with far‐red (WL + FR). We gathered phenotypic data for both MM and M82 at 21 days after germination (dag), as shown in Figure 1a. We observed multiple phenotypic responses induced by WL + FR, with stem elongation being particularly prominent (Figure 1b). We chose the first internode for detailed cellular anatomy phenotyping, as it was the only internode present at the onset of FR treatment (14 days after sowing) and showed the strongest response. The cross and longitudinal sections were prepared at the internode's midpoint, and a set of cell types (Figure 1c) were characterized for their cell layer numbers, areas, lengths, and widths. We discovered many cellular responses to FR (Figures 1d–e), such as interfascicular cambium thickness, pith layer, middle pith length, pith thickness, parenchyma layer, collenchyma layer, collenchyma cell area, and epidermis cell length (*p* < 0.05 in WL vs. WL + FR, Figure 1e). These findings provide a detailed understanding of the cellular changes in tomato plants in response to FR light treatments. As reported in *Arabidopsis*, FR‐induced elongation occurs in the petiole epidermis, and we observed a similar response in tomato. However, pith cells displayed both elongation and pronounced increase in cell layer number—a less explored phenomenon—prompting us to focus our investigation on this cell type.

### 3.2 Shade Avoidance‐Related TFs Identification by RNA‐Seq

**FIGURE 2:**
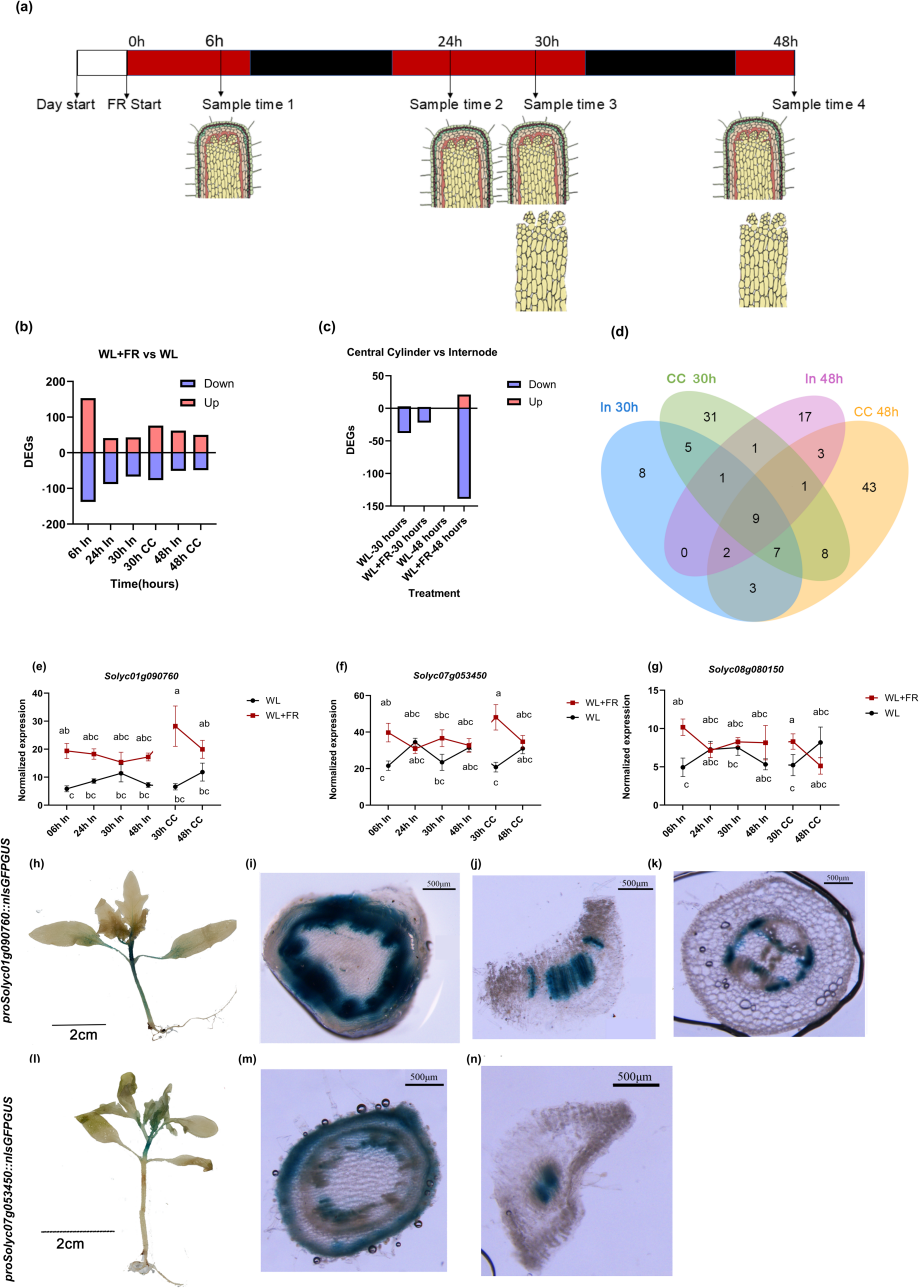
Transcriptomic responses of tomato first internode to low R:FR. (a) The experimental design of the transcriptomics experiment, including the timing of harvest and the specific tissues harvested. White bar represents the WL treatment, red bars represent periods when the supplemental FR light is added, while black bars indicate night phases, and alternating light/dark bars depict the light/dark cycle. (b) DEG analysis of FR‐responsive genes for each timepoint for whole internode and central cylinder samples. Upregulated genes are marked in red and downregulated genes in blue. (c) DEG analysis of central cylinder‐enriched and cylinder‐depleted genes, as identified by DE analysis for central cylinder versus whole internode for specific timepoints and light treatments. Central cylinder‐enriched genes marked in red and central cylinder‐depleted genes in blue. (d) Overlap of FR‐responsive upregulated DEGs between the central cylinder and whole internode at 30 and 48 h of light treatment. (e–g) The expression patterns of the three genes encoding for TFs of interest. (e) *Solyc01g09760*, (f) *Solyc07g053459*, and (g) *Solyc08g080150*. The significant differences are determined using two‐way ANOVA, and error bars represent standard errors. (h–k) GUS staining of 2‐week‐old *proSolyc01g090760::nlsGFPGUS* seedling with 6‐h FR treatment. Cross sections show (i) the first internode, (j) petiole of the first true leaf, and (k) the hypocotyl. (l–n) GUS staining of 2‐week‐old *proSolyc07g053450::nlsGFPGUS* seedling with 6‐h FR treatment. Cross sections show (m) the first internode and (n) petiole of the first true leaf.

We designed an RNA‐seq experiment with four timepoints (6, 24, 30, and 48 h after the start of the light treatment) and harvested the first internode or its central cylinder from tomato stems for RNA extraction. This approach allowed us to identify the gene expression changes accompanying the internode elongation and the pith responses (Figure 2a). We selected timepoints that led to the discernible change in internode elongation (6 h, based on Li et al. 2024). In addition to harvesting whole internode tissue, we were able to hand‐dissect the central cylinder in plants of the latter two timepoints. These central cylinder samples are largely composed of pith but likely include some of the vascular tissues as well. We compared WL + FR samples at each timepoint to the corresponding WL samples to discern the FR‐responsive differentially expressed genes (Figure 2b). Notably, at the 6‐h mark, the most DEGs were observed in the whole internode (153 up, 138 down), followed by a general trend of decreasing DEGs over time (to 62 up, 51 down at 48 h). The central cylinder followed a similar pattern, with more DEGs at 30 h (76 up, 77 down) than at 48 h (50 up, 49 down). Next, we tested the gene expression of the central cylinder against the whole internode for each treatment and timepoint (Figure 2c) and found 26 central cylinder‐enriched genes. To delve deeper, we also intersected the upregulated DEGs between both the central cylinder and the internode at 30 and 48 h, finding a higher count of unique DEGs in the central cylinder (Figure 2d). These are likely central cylinder‐specific responses that are diluted out in the entire internode samples.

We tested the FR‐responsive central cylinder DEGs for GO term enrichment (Figure S2) to see if it resembled that of whole internode (Li et al. 2024), but we identified only a small number of GO terms. GO category “response to auxin” was enriched in the upregulated DEGs in the central cylinder as expected, but surprisingly, only at the 30‐h timepoint and not at 48 h. No other enriched GO terms were shared between FR‐responsive DEGs of whole internode (Li et al. 2024) and central cylinder (Figure S2). We also tested GO term enrichment of the central cylinder‐enriched DEGs (Figure S3) for each timepoint and treatment, and GO category “transmembrane transport” was enriched throughout the central cylinder‐enriched DEGs, either reflecting the little understood roles of pith in transporting molecules or the effect from the residual vascular tissues with known transport roles included in the sampling.

We further examined the DEG lists and identified 46 FR‐responsive TF encoding genes in our data (Table 1). TFs typically coordinate and amplify responses to environmental cues, so we focused our follow‐up inquiry on selected TFs of interest. The dataset contains 10 bHLH (Basic helix–loop–helix) TFs, 8 MYB TFs, 4 basic‐leucine zipper TFs, and 3 homeobox‐leucine zipper‐like TFs. Specifically, we selected three upregulated TFs for detailed analysis based on their functions reported in the literature. These TFs, encoded by *Solyc08g080150*, *Solyc01g090760*, and *Solyc07g053450*, showed upregulation in low R:FR in both the whole internode but also in the central cylinder (Figure 2e–g).

**TABLE 1:** **DEGs that are transcriptome factors.** Functional annotations were obtained from Plant Transcription Factor Database (Jin et al. 2015, 2017), Sol genomics network (Fernandez‐Pozo et al. 2015) and literature (Yang et al. 2015; Padmanabhan et al. 2022). Homologs from *Arabidopsis* were identified by multi‐blast using *Arabidopsis* protein sequences.

DEGs list	altName	Function annotation from database	TAIR_ID	Short description_(30‐06‐2021)	Curator_summary_(30‐06‐2021)
Solyc04g007000	AP2/B3 transcription factor family protein	Ethylene‐responsive transcription factor4	AT1G13260	Related to ABI3/VP1 1	Encodes an AP2/B3 domain transcription factor, which is upregulated in response to low temperature. It contains a B3 DNA‐binding domain. It has diurnal regulation and may function as a negative growth regulator. The mRNA is cell‐to‐cell mobile
Solyc07g005400	bHLH transcription factor 050	Regulation of cell size	AT2G24260	LJRHL1‐like 1	Encodes a basic helix–loop–helix (bHLH) protein that regulates root hair and sperm cell development. One of the three *Arabidopsis* homologs of the *Lotus japonicus* ROOTHAIRLESS1 (LjRHL1) gene: At2g24260 (AtLRL1), At4g30980 (AtLRL2), and At5g58010 (AtLRL3)
Solyc08g080150	TCP transcription factor 20	Regulation of cell size	AT2G43060	TCP family transcription factor	—
Solyc01g090760	GATA transcription factor		AT2G45050	GATA transcription factor 2	Encodes a member of the GATA factor family of zinc finger transcription factors. A positive regulator of photomorphogenesis
Solyc04g077780	LIM domain‐containing protein		AT1G10200	GATA type zinc finger Transcription factor family protein	Encodes a member of the *Arabidopsis* LIM proteins: a family of actin bundlers with distinct expression patterns. WLIM1, WLIM2a, and WLIM2b are widely expressed, whereas PLIM2a, PLIM2b, and PLIM2c are predominantly expressed in pollen. Regulates actin cytoskeleton organization
Solyc10g079070	bHLH transcription factor 065		AT3G57800	Basic helix–loop–helix (bHLH) DNA‐binding superfamily protein	Together with bHLH48 associates with phytochrome interacting factor 7 to regulate hypocotyl elongation
Solyc07g053450	Basic‐leucine zipper (BZIP) transcription factor family protein	Leaf cell number and cell size	AT2G42380	Basic‐leucine zipper (bZIP) transcription factor family protein	Encodes a member of the BZIP family of transcription factors. Forms heterodimers with the related protein AtbZIP61. Binds to G‐boxes in vitro and is localized to the nucleus in onion epidermal cells
Solyc05g009660	Transcription factor PERIANTHIA	Regulation of flower development	AT1G68640	bZIP transcription factor family protein	Encodes bZIP transcription factor. Mutant plants have extra floral organs. PAN is essential for AG activation in early flowers of short‐day‐grown plants. Binds directly to 5′‐AAGAAT‐3′regulatory sequence in AG promoter
Solyc05g048830	MYB transcription factor		AT3G61250	myb domain protein 17	LATE MERISTEM IDENTITY2 (LMI2) is a target of the meristem identity regulator LEAFY (LFY). Has a role in the meristem identity transition from vegetative growth to flowering. Member of the R2R3 factor gene family
Solyc06g049040	Basic‐leucine zipper (BZIP) transcription factor family protein		AT2G40620	Basic‐leucine zipper (bZIP) transcription factor family protein	Basic‐leucine zipper transcription factor. Localizes from cytoplasm to the nucleus under heat stress
Solyc07g043580	bHLH transcription factor 052	Deetiolation; red light signaling pathway; response to low‐fluence blue light stimulus by blue low‐fluence system; regulation of auxin biosynthetic process	AT2G43010	Phytochrome interacting factor 4	Isolated as a semidominant mutation defective in red light responses. Encodes a nuclear‐localized bHLH protein that interacts with active PhyB protein. Negatively regulates phyB‐mediated red light responses. Involved in shade avoidance response. Protein abundance is negatively regulated by PhyB. Involved in the regulation of response to nutrient levels. Controls the resistance to *B. cinerea* in a COI1‐ and EIN2‐dependent manner
Solyc01g109700	bHLH transcription factor 010	Response to blue light; positive regulation of flower development	AT4G34530	Cryptochrome‐interacting basic helix–loop–helix 1	Encodes a transcription factor CIB1 (cryptochrome‐interacting basic helix–loop–helix). CIB1 interacts with CRY2 (cryptochrome 2) in a blue light‐specific manner in yeast and *Arabidopsis* cells, and it acts together with additional CIB1‐related proteins to promote CRY2‐dependent floral initiation. CIB1 positively regulates FT expression
Solyc05g055940	Vacuolar protein sorting‐associated protein (DUF946)		AT5G29000	Homeodomain‐like superfamily protein	MYB‐CC family member. PHL1 acts redundantly with PHR1 to regulate responses to Pi starvation
Solyc06g083170	bHLH transcription factor 049		AT3G07340	Basic helix–loop–helix (bHLH) DNA‐binding superfamily protein	—
Solyc09g014250	Transcription factor DIVARICATA		AT2G38090	Duplicated homeodomain‐like superfamily protein	—
Solyc02g076640			AT4G18650	Transcription factor‐related	A maternally expressed imprinted gene in the endosperm. Its expression is positively regulated by ROS1
Solyc02g078130		Regulation of transcription from RNA polymerase II promoter; RNA polymerase II transcription factor complex; defense response to fungus	AT4G20970	Basic helix–loop–helix (bHLH) DNA‐binding superfamily protein	—
Solyc03g006910			AT4G25410	Basic helix–loop–helix (bHLH) DNA‐binding superfamily protein	—
Solyc04g005660			AT3G28857	Basic helix–loop–helix (bHLH) DNA‐binding family protein	Encodes an atypical member of the bHLH (basic helix–loop–helix) family transcriptional factors
Solyc04g007430			AT1G09530	Phytochrome interacting factor 3	Transcription factor interacting with photoreceptors phyA and phyB. Forms a ternary complex in vitro with G‐box element of the promoters of LHY, CCA1. Acts as a negative regulator of phyB signaling. It degrades rapidly after irradiation of dark grown seedlings in a process controlled by phytochromes. Does not play a significant role in controlling light input and function of the diurnal clockwork. Binds to G‐ and E‐boxes, but not to other ACEs. Binds to anthocyanin biosynthetic genes in a light‐ and HY5‐independent fashion. PIF3 function as a transcriptional activator can be functionally and mechanistically separated from its role in repression of PhyB‐mediated processes
Solyc06g062460			AT3G21330	Basic helix–loop–helix (bHLH) DNA‐binding superfamily protein	—
Solyc07g008010	R2R3MYB transcription factor 82	Trichome differentiation	AT5G52600	myb domain protein 82	Encodes a nuclear‐localized transcription activator that is a member of the R2R3 factor gene family. MYB82 and GL1 can form homodimers and heterodimers at R2R3‐MYB domains. At least one of the two introns in MYB82 is essential to the protein's trichome developmental function
Solyc07g056360			AT2G42870	Phy rapidly regulated 1	Encodes PHYTOCHROME RAPIDLY REGULATED1 (PAR1), an atypical basic helix–loop–helix (bHLP) protein. Closely related to PAR2 (At3g58850). Up regulated after simulated shade perception. Acts in the nucleus to control plant development and as a negative regulator of shade avoidance response. Functions as transcriptional repressor of auxin‐responsive genes SAUR15 (AT4G38850) and SAUR68 (AT1G29510)
Solyc09g065100	bHLH transcription factor 150	Trichome differentiation; seed coat development; regulation of proanthocyanidin biosynthetic process	AT4G09820	Basic helix–loop–helix (bHLH) DNA‐binding superfamily protein	TT8 is a regulation factor that acts in a concerted action with TT1, PAP1 and TTG1 on the regulation of flavonoid pathways, namely, proanthocyanidin and anthocyanin biosynthesis. Affects dihydroflavonol 4‐reductase gene expression. It is thought that a ternary complex composed of TT2, TT8, and TTG1 is necessary for correct expression of BAN in seed endothelium. Also important for important for marginal trichome development. It binds the promoter of both AT3G26790 and AT1G28300.TT8 interacts with JAZ proteins to regulate anthocyanin accumulation. TT8 acts maternally to affect seed FA biosynthesis and inhibits seed FA accumulation by downregulating a group of genes either critical to embryonic development or important in the FA biosynthesis pathway. TT8 represses the activities of LEAFY COTYLEDON1, LEAFY COTYLEDON2, and FUSCA3, the critical transcriptional factors important for seed development
Solyc10g052470	Myb family transcription factor (Fragment)		AT4G39250	RAD‐like 1	—
Solyc10g076820	Myb family transcription factor		AT2G38090	Duplicated homeodomain‐like superfamily protein	—
Solyc10g084340			AT2G28550	Related to AP2.7	AP2 family transcription factor that is involved in regulation of flowering and innate immunity. Interacts with CRY2 to regulate CO and FT. TOE1 binds to activation domain of CO and binds CORE sequences of the FT promoter. TOE1/TOE2 are also targets of MiR172b and function in regulation of innate immunity
Solyc10g086250	R2R3MYB transcription factor 75		AT1G66370	myb domain protein 113	Encodes a member of the MYB family of transcription factors. Involved in regulation of anthocyanin biosynthesis. Affects the expression of enzymes involved in later steps of anthocyanin biosynthesis
Solyc12g008800	MYB transcription factor		AT5G56840	myb‐like transcription factor family protein	—
Solyc12g010800	BZIP transcription factor family protein	Leaf cell number and cell size	AT3G58120	Basic‐leucine zipper (bZIP) transcription factor family protein	Encodes a member of the BZIP family of transcription factors. Forms heterodimers with the related protein AtbZIP34. Binds to G‐boxes in vitro and is localized to the nucleus in onion epidermal cells
Solyc12g049350	R2R3MYB transcription factor 11		AT2G47460	myb domain protein 12	MYB12 belongs to subgroup 7 of the R2R3‐MYB family. It strongly activates the promoters of chalcone synthase (CHS), flavanone 3‐hydroxylase (F3H), flavonol synthase (FLS), and—to a lesser extent—chalcone flavanone isomerase (CHI), but cannot activate the promoters of flavonoid‐3′hydroxylase (F3′H) and dihydroflavonol 4‐reductase (DF). The activation requires a functional MYB recognition element (MRE). Results from the myb12‐1f allele indicate that an activation domain might be present in the C‐terminus. Overexpression or knockout plants do not show any obvious phenotype under greenhouse conditions. Young myb12‐ko seedlings contain reduced amounts of flavonoids (quercetin and kaempferol), while seedlings as well as leaves of MYB12‐OX plants displayed an increased flavonoid content. They did not show any significant difference in anthocyanin content. Expression of CHS and FLS shows a clear correlation to MYB12 expression levels. CHI and F3H show increased transcript levels in the MYB12‐OX lines, but no differences in the knockout. Even in the absence of functional MYB12, flavonol biosynthesis is not completely absent, suggesting functional redundancy. The redundant factors are MYB11 and MYB111 although MYB12 is primarily required for flavonol biosynthesis in roots. Mutations in MYB12 block both auxin and ethylene stimulation of flavonoid synthesis
Solyc01g010910	MYB transcription factor	Lateral organ boundaries	AT2G03810	18S pre‐ribosomal assembly protein gar2‐like protein	
Solyc01g090460	HD‐ZIP	Induction: By transition from light to far‐red light. {ECO:0000269|PubMed:8106086}	AT1G80810	One of 5 PO76/PDS5 cohesion cofactor orthologs of *Arabidopsis*	Involvement of the cohesin cofactor PDS5 (SPO76) during meiosis and DNA repair in *Arabidopsis thaliana*
Solyc01g091630	Cutin deficient 2	Regulation of transcription, DNA‐templated; plant‐type cell wall modification; cuticle development; anthocyanin accumulation in tissues in response to UV light; root hair cell differentiation	AT4G00730	AHDP, ANL2, anthocyaninless 2, *Arabidopsis thaliana* homeodomain protein	Encodes a homeodomain protein of the HD‐GLABRA2 group. Involved in the accumulation of anthocyanin and in root development. Loss of function mutants has increased cell wall polysaccharide content
Solyc01g102340	R2R3MYB transcription factor 61	DNA‐binding, regulation of transcription	AT1G74440	MHL	Similar to MPH1, can complement mph1–1 salt sensitivity phenotype
Solyc01g107190	LOB domain‐containing protein 38	Lateral organ boundaries	AT2G03810		18S pre‐ribosomal assembly protein gar2‐like protein, regulation of asymmetric cell division
Solyc02g077590	Homeobox‐leucine zipper‐like protein	Regulation of transcription, DNA‐dependent; response to blue light and the absence of light	AT3G59010	PECTIN METHYLESTERASE 35, PECTIN METHYLESTERASE 61, PME35, PME61	Encodes PME35, a pectin methylesterase. PME35‐mediated demethylesterification of the primary cell wall regulates the mechanical strength of the supporting tissue; involved in cell wall modification, pectin catabolic process
Solyc02g080260	Woolly	Binding to specific DNA	AT4G00730	ANTHOCYANINLESS 2	Encodes a homeodomain protein of the HD‐GLABRA2 group. Involved in the accumulation of anthocyanin and in root development. Loss of function mutants has increased cell wall polysaccharide content
Solyc02g081270	NAC domain‐containing protein 53	Regulation of transcription	AT3G56900	ALADIN	Encodes ALADIN, a component of the nuclear pore complex
Solyc02g086930	Homeobox‐leucine zipper protein HAT5	Sequence‐specific DNA binding	AT2G44230	Hypothetical protein	
Solyc02g087960	R2R3MYB transcription factor 94	Sequence‐specific DNA‐binding transcription factor activity	AT1G76990	ACT domain repeat 3	
Solyc02g088190	MYB transcription factor	Sequence‐specific DNA‐binding transcription factor activity	AT1G74440	Similar to MPH1, can complement mph1–1 salt sensitivity phenotype	Cellular response to hormone stimulus, cellular response to lipid, cellular response to oxygen‐containing compound, response to osmotic stress, response to water, and signal transduction
Solyc02g089420	Basic‐leucine zipper 43	Protein heterodimerization activity	AT5G52170	HOMEODOMAIN GLABROUS 7	Encodes a homeobox‐leucine zipper family protein belonging to the HD‐ZIP IV family
Solyc02g090400	Myb family transcription factor	Regulation of transcription, DNA‐templated; specification of organ identity; negative regulation of gene expression; floral organ formation	AT5G24400	6‐PHOSPHOGLUCONOLACTONASE 3, EMB2024, EMBRYO DEFECTIVE 2024, PGL3	Encodes a protein with 6‐phosphoglucunolactonase activity that localizes to the chloroplasts and the peroxisome. However, mutant phenotypes observed in pgl3 mutant plants can be complemented with a chloroplast‐targeted version of the protein. PGL3 likely functions in the oxidative branch of the pentose phosphate pathway. pgl3 mutant phenotypes suggest that it is important in pathogen defense and maintenance of cellular redox homeostasis
Solyc03g007410	Basic helix–loop–helix (BHLH) DNA‐binding superfamily protein	Stomatal complex development	AT5G07770	Actin‐binding FH2 protein	
Solyc03g082430	Growth‐regulating factor 5	regulation of transcription, DNA‐template; response to red light; response to far‐red light; response to Karrikin	AT2G45480	ATGRF9, GRF9, GROWTH‐REGULATING FACTOR 9	Growth‐regulating factor encoding transcription activator. One of the nine members of a GRF gene family, containing nuclear targeting domain. Involved in leaf development

*Solyc01g090760* encodes for a TF from the zinc finger DNA‐binding protein family, with roles for development and cell differentiation (Gao et al. 2015). Its *Arabidopsis* homologs are implicated in longitudinal cell elongation, linking well with the stem and the cell elongation responses observed (Lu et al. 2021; Hou et al. 2022). *Solyc08g080150* encodes for SlTCP20, a TF with diverse roles in leaf development, flower symmetry regulation, shoot branching, and senescence involvement (Parapunova et al. 2014). Lastly, *Solyc07g053450* codes for a bZIP TF, which is integral to several biological functions, notably in the response to various abiotic stresses (Liu et al. 2023b).

We wanted to test the spatial expression patterns driven by the promoters of these TFs. We constructed transcriptional reporter lines for *Solyc01g090760* and *Solyc07g053450*, while we were not able to regenerate tomato lines with the *Solyc08g080150* transcriptional reporter construct. We visualized their expression with GUS staining, and this revealed distinct expression patterns for two TFs in tomato (Figure 2h–n). The *Solyc01g090760* promoter showed abundant expression primarily in the hypocotyl, the first internode, and the petiole of true leaves. Detailed histological analysis narrowed down this expression to the xylem of the hypocotyl and first internode, as well as to young pith cells, and the central vascular bundle of the petiole. *Solyc07g053450* demonstrated specific expression in the internode and the petiole of true leaves, while conspicuously absent in the hypocotyl. Closer examination of cross sections revealed its localization to the epidermis and young pith cells of the internode, and strictly to the stele, excluding the phloem, in the petiole. This differential expression suggests a role for Solyc07g053450 in regulating tissue‐specific developmental processes, potentially influencing structural and physiological traits in these regions.

These findings provide insights into the spatial regulation of gene expression during early plant development, highlighting the intricate roles these TFs may play in tomato morphogenesis.

### 3.3 Pith Elongation Is a Conserved Behavior in Shade‐Avoiding Species

**FIGURE 3:**
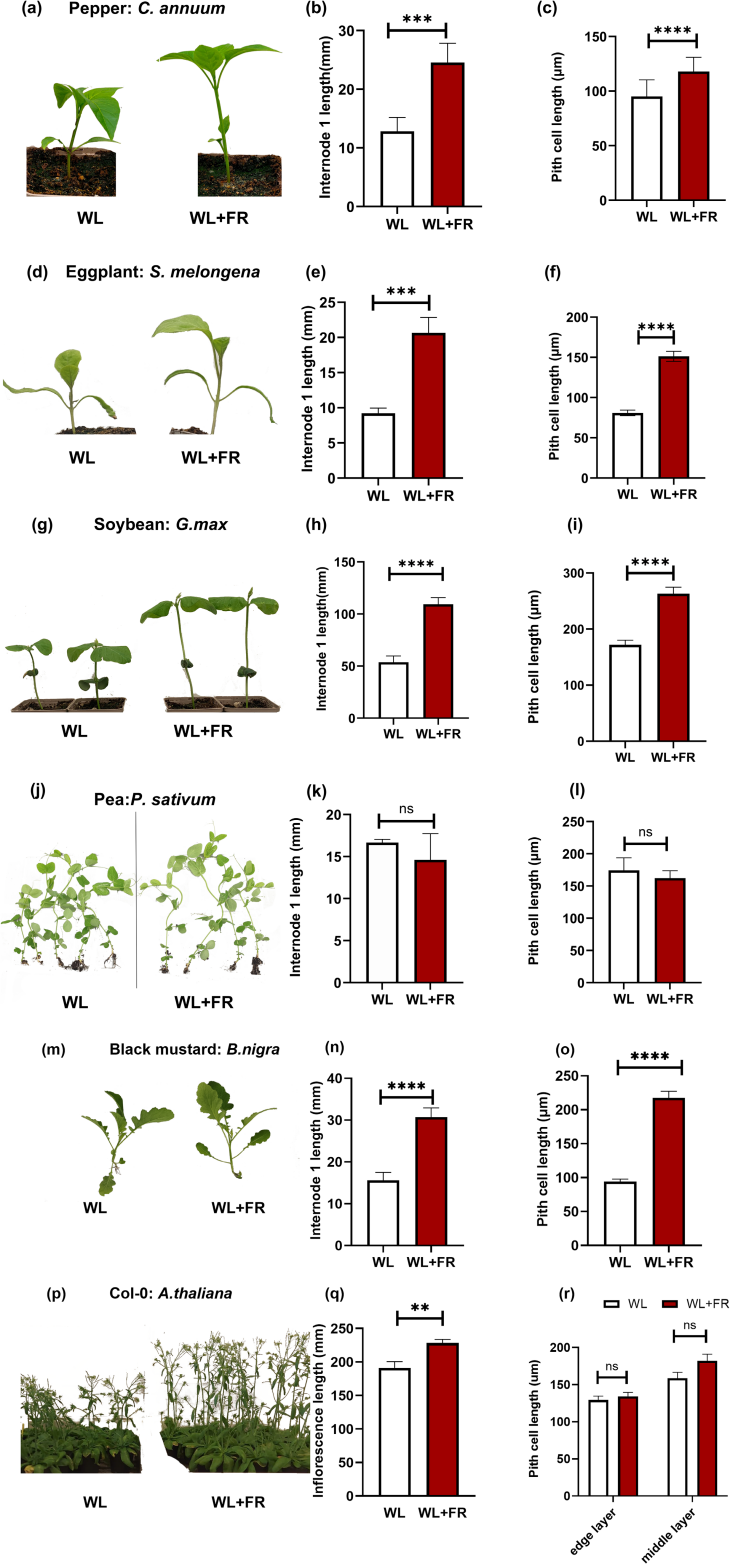
FR‐responsive internode elongation is accompanied with pith cell changes across multiple dicots. Representative images of 7‐day WL and WL + FR‐treated plants (a,d,g,j,m,p), measurements of the first internode or inflorescence length (b,e,h,k,n,w), and pith cell length measurements (c,f,i,l,n,r). The data are given by species as follows: (a–c) bell pepper ( *Capsicum annuum* ), (d–f) eggplant ( *Solanum melongena* ), (g–i) soybean ( *Glycine max* ), (j–l) pea ( *Pisum sativum* ), (m–o) black mustard ( *Brassica nigra* ), and (p–r) *Arabidopsis* (Col‐0). Asterisks in the figure denote significant differences between WL and WL + FR conditions, with: * represents *p* ≤ 0.05, ** *p* ≤ 0.01, *** *p* ≤ 0.001, and **** *p* ≤ 0.0001. The sample size for each phenotyping comparison was approximately 12, and each experiment was repeated twice. The sample size for each cellular phenotyping comparison was approximately 50.

After observing the cellular response to FR enrichment in tomato, we wondered if the pith phenotypes and specific transcriptional changes were limited to tomato, or if these responses were conserved across different dicot species with similar stem growth habits. We included two other Solanaceae family members, *C. annuum* (bell pepper) and *S. melongena* (eggplant). For more distantly related species, we chose two pairs of species from the rosid clade. Firstly, we chose soybean ( *G. max* ) and *P. sativum* (pea), from the Fabaceae (legume) family and *B. nigra* (black mustard) and *Arabidopsis* from the Brassicaceae family. However, *Arabidopsis* does not form elongated internodes during juvenile growth, so there we looked into the inflorescence stem responses instead to test if this could serve as a model for stem responses. In these species, we observed a marked elongation response in internode 1 under supplemental FR light exposure (Figure 3). We also measured stem diameter and hypocotyl traits, and in some species, the elongation was accompanied by diameter increase (Figure S4). A common characteristic among these species, except for the *Arabidopsis* inflorescence, was the elongation of pith cells in the internode 1 (Figure 3). These findings establish tomato, bell pepper, eggplant, black mustard, and soybean as examples of FR‐responsive species in our panel. Conversely, when testing pea, we noted no significant differences in stem length between the treatments (Figure 3), concluding that it is a species with lesser FR response. We also noted that the cellular arrangement of pea internodes was different to the other species we investigated (Figure S5). Cross sections of pea stems revealed a tetrarch symmetry, creating an X‐shaped pith, thus requiring specific directions for measurement (Figure S5). Here, to measure pith traits, we focused on the area predominantly composed of densely red‐stained cells within the X‐shaped area between vascular bundle. Our analysis showed no differences between treatments for the measured cell types in pea (Figure 3l), highlighting the variability in FR‐responsiveness among different dicot species.

### 3.4 FR‐Responsive Tomato TFs Have Family‐Specific and Tissue‐Specific Expression Patterns

Finally, we wanted to test if the molecular signatures coupled with internode and pith elongation responses to FR light in tomatoes were conserved in our set of dicots. Hence, we tested if the FR‐responsive behavior of the transcripts for our candidate TFs identified in the tomato transcriptomics data, *Solyc07g053450, Solyc08g080150*, and *Solyc01g090760*, was also observed in the other species.

**FIGURE 4:**
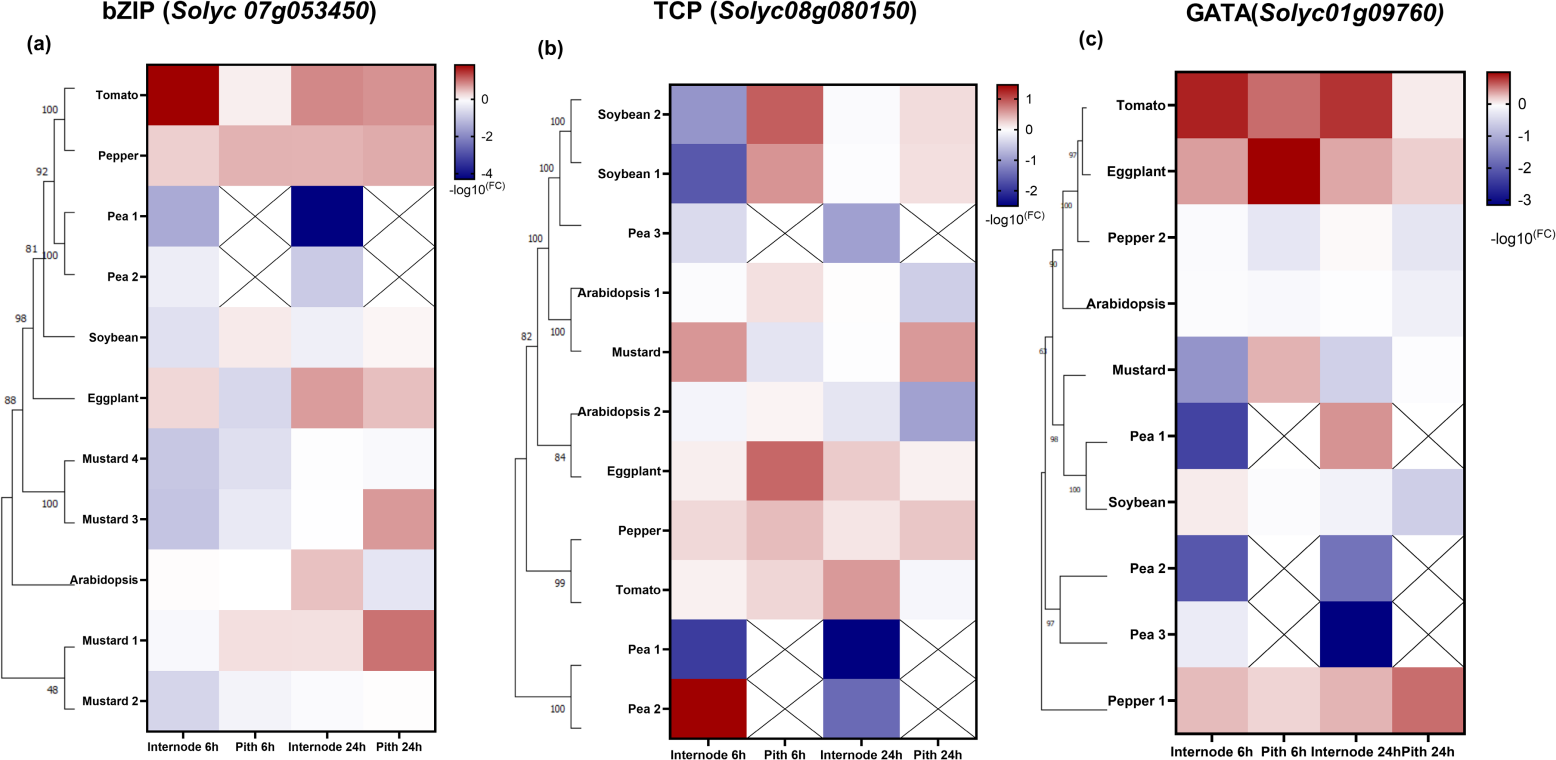
FR‐responsiveness of target TF expression is conserved in Solanaceae. (a) Phylogeny and expression patterns of *Solyc07g053450* homologs: tomato (*Solyc07g053450*), bell pepper (*CA00g71840:1‐921*), eggplant (*SMEL4.1_07g019740.1.01*), *Arabidopsis* (*AT2G42380*), four black mustard homologs *(BniB06g001480.2N.1*, *BniB08g027470.2N.1*, *BniB08g072820.2N.1*, *BniB06g055220.2N.1*), soybean (*Gm06:49537899..49539195*), and two pea homologs (*XM_051027770.1*, *XM_051045459.1*). (b) Phylogeny and expression patterns of *Solyc08g080150* homologs: tomato (*Solyc08g080150*), bell pepper (*TCP19 XM_016712397.2*), eggplant (*chr8:85852001‐85853900*), two *Arabidopsis* homologs (*AT2G45680*, *AT5G51910*), black mustard (*BniB08g029800.2N.1*), two soybean homologs (*Glyma.07G080300.1*, *Glyma07g08710.2*), and two pea homologs (*XM_051030490.1*, *XM_051030491.1*). (c) Phylogeny and expression patterns of *Solyc01g090760* homologs: tomato (*Solyc01g090760*), two bell pepper homologs (*chr3:267174801‐267176100*, *chr8:35902201‐35904000*), eggplant (*chr8:35902201‐35904000*), *Arabidopsis* (*AT2G45050*), black mustard (*BniB07g039570.2N.1*), soybean (*GlysoPI483463.06G078800.1*), and three pea homologs (*XM_051027377*, *XM_051030552.1*, *XM_051038772.1*). Each section utilizes Mega X software for phylogenetic analysis, adopting the Tamura‐Nei model with a bootstrap analysis (1000 replicates) for enhanced accuracy. The gene expression data from qRT‐PCR is represented as heatmaps of log fold changes in gene expression under WL + FR versus WL conditions in the internode or pith at 6 and 24 h after FR treatment, with red indicating upregulation, blue for downregulation, and white for no change (logFC = 0).

For this analysis, we identified homologs for the three TF genes in all these species using BLAST (Fernandez‐Pozo et al. 2015; The Arabidopsis Information Resource 2015; Chen et al. 2022). In Solanaceae bell pepper and eggplant, we identified single hits for each of the three genes, whereas for *Arabidopsis*, homolog selection was guided by BLAST results and literature annotations to identify a homolog with a potentially conserved function. For pea, soybean, and black mustard, we chose genes based on high similarity scores (> 80%). These homologs were visualized in maximum likelihood phylogenies (Figure 4), which generally reflected the relationships between the plant species and indicated recent gene or whole‐genome duplication events. To quantify the FR‐responsive gene expression, we collected samples from whole first internodes and their central cylinder at 6‐ and 24‐h timepoints for WL and WL + FR light treatments. For pea, the central cylinder could not be dissected due to challenging morphology (Figure S5).

The FR‐responsive upregulation of *Solyc07g053450* and *Solyc08g080150* orthologs was consistent in both internode and central cylinder across the Solanaceae family: tomato, eggplant, and bell pepper (Figures 4, S6, S7). For *Solyc01g090760*, the conserved FR‐induced upregulation was observed in tomato and eggplant, while in bell pepper, there were two homologs that showed divergent expression patterns. Unexpectedly, the bell pepper homolog that was less similar to *Solyc01g090760* was FR‐induced, while the homolog that was more similar had lost its FR‐responsiveness (Figures 4, S8). This indicates that even with high sequence similarity, homologs can have diverged function, especially if another homolog has retained its role.

Outside the Solanaceae family, in *Arabidopsis*, black mustard, pea, and soybean, we generally observed a downregulation of these TF homologs in response to FR supplementation (Figure 4). In pea, where limited stem growth under FR was noted, all three TF homologs consistently showed decreased expression in response to FR. This suggests that these TFs might have acquired FR‐responsive behavior within the Solanaceae lineage or lost it in the pea lineage. In soybean, which exhibited a less clear downregulation of the transcripts, the soybean homologs of *Solyc08g080150* showed strong FR‐induction in the pith at 6 h (Figure 4), indicating some conservation of function. In the FR‐responsive soybean, while there is not such a clear downregulation in FR, there is also no clear homolog that would match the tomato patterns. For the two soybean homologs of *Solyc08g080150,* we observed a strong FR‐induction specifically in central cylinder at 6 h (Figure 4), so potentially, the role of these two homologs is retained to some extent in soybean.

Thus, we observed that the expression patterns of FR‐responsive genes were similar within the Solanaceae family but differ significantly from those in the Brassicaceae and Fabaceae families. Although certain species in Brassicaceae and Fabaceae showed elongation responses akin to those observed in Solanaceae, the expression patterns of the TFs were not conserved across all families.

## 4 Discussion

While extensive studies have been conducted on the role of the epidermis in SAS in *Arabidopsis* (Kohnen et al. 2016; Procko et al. 2016; Küpers et al. 2023), there is a notable gap in our understanding of the role of other cell types and especially that of pith. The distinct anatomical and functional aspects of tomato stems and *Arabidopsis* petioles highlight this discrepancy. The pith, centrally located in the stem, is crucial for plant physiology and morphology but is often overlooked. This paper focuses on the pith responses and relates them to SAS across multiple species.

### 4.1 FR‐Induced Cell Elongation, Predominantly Occurring in the Pith in Tomato, Is Likely Conserved but With Some Exceptions

SAS has been extensively studied in *Arabidopsis*, where elongation traits in the hypocotyl and petiole show similarities to those seen in tomatoes (Chitwood et al. 2015). Research in *Arabidopsis* has highlighted a crucial aspect of SAS: the involvement of xyloglucan endotransglucosylase/hydrolase (XTH) proteins in cell wall relaxation, which aids cell expansion under shaded conditions (Sasidharan et al. 2010, 2014). This mechanism is instrumental in petiole elongation when exposed to supplemental FR light (Sasidharan et al. 2010). Specifically, the epidermis cells in the hypocotyl (18 days) (Procko et al. 2016) and petiole (Pantazopoulou et al. 2017) of *Arabidopsis* become longer in response to supplemental FR light. In fact, the epidermis acts as a signal integrator in *Arabidopsis* and regulates organ elongation (Brown et al. 1995; Savaldi‐Goldstein and Chory 2008). However, the tissue morphologies and growth habits of *Arabidopsis* and tomato differ, making direct comparisons challenging. One of the differences between the cellular morphology of *Arabidopsis* petiole and tomato stem is the absence of pith cells in the *Arabidopsis* petiole. Pith cells, known as spongy parenchyma cells, provide support and nutrient storage in stems and roots. They are known for their extensibility, which differs from the outer layers (Abercrombie et al. 1968; Gallego‐Giraldo et al. 2016; Yang et al. 2016). Additionally, pith undergoes programmed cell death as the tomato stem matures (Esau 1953; Fujimoto et al. 2018). When we characterized the cellular anatomy responses of tomato cultivars M82 and MM to supplemental FR, we found a pronounced response of the pith to FR enrichment, with increased pith cell layers and cell length (Figure 1). While epidermis cells also became longer, their response was less pronounced compared to the pith. This led to a further investigation into how these cell types perceive and mediate SAS signals.

We profiled the FR‐responsive gene expression patterns in the central cylinder of two tomato cultivars, M82 and MM (Figure 2). The central cylinder is largely composed of pith cells, but given the restrictions of hand dissection, some vascular cells are also likely to be present. We observed a notable increase in auxin‐responsive genes, especially SAURs, under low R:FR conditions, with diurnal rhythm playing a key regulatory role (Li et al. 2024). The examination of central cylinder‐specific transcriptomes revealed enrichment of “response to auxin” in the upregulated DEGs at 30 h, but not at 48 h. As the whole internode upregulated DEGs demonstrated enrichment of “response to auxin” that built up along the time course to 48 h, this may be reflective of how auxin reaches the various tissues, likely inward out, and how the response builds up. It also may indicate that the auxin response is shorter lived in the central cylinder than in the outer tissue layers. We recently showed in Li et al. (2024) that the role of auxin in FR‐responsive internode elongation in tomato is more complicated than in *Arabidopsis*, as exogenous indole‐3‐acetic‐acid (IAA) treatments were not able to recreate the full FR‐induced elongation response, and we did not observe a FR‐responsive increase in IAA in the internode. Taken together, this may support the importance of the tissue‐specific distribution of auxin and its signaling. Additionally, the central cylinder demonstrated other significant differences from the whole internode, highlighting specific regulatory pathways (Figure S1). The involvement of various TFs in low R:FR response was evident, leading us to investigate these TFs further.

We then explored how conserved these responses were. We started with phenotyping of supplemental FR internode and pith responses in bell pepper, pea, soybean, eggplant, black mustard*,* and *Arabidopsis* (Figure 3). We observed that most species shared responses with tomato; FR‐responsive internode elongation accompanied by increased pith cell length. Exceptions were pea with mild or no internode response and no cellular elongation (Ko et al. 2004; Skubisz et al. 2007). Pea photoreceptors, while sharing many similarities with those of other plants, exhibit unique traits such as the duplication of the CRY2 gene—which shows differential regulation under various light conditions—and novel mutants like lfp1‐1 (late‐flowering photomorphogenic mutant) that display distinct phenotypes under blue light, highlighting differences in how peas integrate light signals compared to other species (Platten 2003). However, further research is needed to fully characterize these differences and their functional implications. The conservation of this response in *Arabidopsis* and other plants is not well documented, but it is known that stem tissue expansion, including pith cells, is influenced by hormonal signals and genetic regulation (Nagata et al. 2004; Ye et al. 2021).

### 4.2 Identification of Tissue‐ and Cell‐Type–Specific FR‐Responsive TFs Unique to Solanaceae

The identification of TFs used in SAS is key to understanding how plants acclimate to variable light conditions. Uncovering these TFs helps understand the regulatory networks guiding the responses in shade‐exposed plants. Additionally, recognizing tissue‐specific expression patterns of these TFs adds power to the discovery, given that different plant tissues may respond uniquely to light variations. In our examination of FR‐induced TF genes in tomatoes (Figure 2e–g), we focused on *Solyc01g090760* (GATA TF), *Solyc08g080150* (TCP TF), and Solyc07g053450 (bZIP TF). We generated promoter reporter gene fusions of two of the TF‐encoding genes, *Solyc01g090760* and *Solyc07g053450*, revealing tissue‐ and cell‐type–specific expression patterns in the internode central cylinder that extended into vasculature of petioles and leaves (Figure 2h–n).

Finally, we compared the FR‐responsiveness of three tomato TF‐encoding genes with their homologs in bell pepper, pea, soybean, eggplant, black mustard*,* and *Arabidopsis* (Figure 4). The observed divergence in FR‐responsive expression patterns between the Solanaceae and the Brassicaceae/Fabaceae families raises compelling questions regarding the genetics and evolutionary aspects of the FR response. This variation leads to a hypothesis that the TF behaviors, characterized by upregulation in Solanaceae and downregulation in Brassicaceae/Fabaceae, might reflect a family‐specific function induced by FR light in these TFs. This scenario opens new paths for investigating distinct regulatory mechanisms governing SAS in the Solanaceae family, as opposed to other diverse plant families. Then, to identify what may be the common responses between these families and the unique FR‐responsive TFs in Fabaceae and Brassicaceae, a wider RNA‐seq profiling targeting species from those families would be an interesting follow‐up.

To contrast our data with that of existing *Arabidopsis* datasets, we utilized the eFP browser to examine *Arabidopsis* gene expression patterns of the TF homologs *AT5G51910, AT2G45050*, *AT2G45680*, and *AT2G42380* (Nakabayashi et al. 2005; Schmid et al. 2005; Winter et al. 2007). For the *Arabidopsis* homologs of *Solyc08g080150* and *AT5G51910*, exhibited increased expression in the inflorescence and seeds while *AT2G45680* showed high expression in leaves and the second internode of the inflorescence stem. For the *Solyc01g090760* homolog *AT2G45050,* peak expression was in the petals. For *AT2G42380*, homolog of *Solyc07g053450*, demonstrated strong expression in mature pollen. These varied expression patterns indicate distinct roles for each homolog in specific developmental stages of *Arabidopsis*. Additionally, *AT2G45050*, *AT2G45680*, and *AT2G42380* have been reported to be upregulated in response to supplemental FR at the *Arabidopsis* leaf tip (Küpers et al. 2023). These genes were also found differentially expressed in the hypocotyl following supplemental FR seedling treatment (Kohnen et al. 2016), highlighting their FR‐responsiveness in *Arabidopsis* tissues beyond those initially profiled in our study. This insight points towards a broader context of FR‐responsive regulation in *Arabidopsis* that may extend beyond the developmental stages and tissues we initially focused on.

The lack of conservation in TF expression across different species suggests that the regulatory pathways activating these TFs have undergone evolutionary divergence, leading to distinct gene expression patterns. Conservation of TF binding events often correlates with the conservation of gene expression at the gene level (Hemberg and Kreiman 2011). However, this conservation can vary significantly across species and tissues (Diehl and Boyle 2018). Thus, the disparity in TF expression across different species implies that the regulatory mechanisms governing these TFs have evolved uniquely, potentially resulting in species‐specific or tissue‐specific expression patterns. This finding reminds us of the complex nature of evolutionary adaptations in plant responses and importance in characterizing traits in different plant families.

### 4.3 Perspectives on the Role of Pith in SAS

Overall, we observed concurrent internode and pith elongation across the species studied, with transcriptionally typically inactive pith showing increased expression of these TFs in Solanaceae. However, our data do not conclusively link the pith function to shade avoidance elongation, and this remains to be firmly established.

Future research directions include extending studies to closely related families of Solanaceae and beyond, to pinpoint when FR‐responsive behavior of these TFs ceases. Functional validation of these TF genes in shade avoidance through knockout and induction experiments would clarify their role. Additionally, investigating plants with varied pith morphologies could offer further insight. Specifically, it would be intriguing to determine if FR‐responsive pith elongation and internode elongation are decoupled in any dicot species, shedding light on the diversity of plant acclimation strategies to light environments.

## Author Contributions

**Kaisa Kajala and Ronald Pierik:** conceptualization and supervision. **Linge Li:** funding acquisition, data curation, formal analysis, visualization, and writing – original draft. **Linge Li, Yorrit van de Kaa, and Lotte van der Krabben:** investigation. **Linge Li, Kaisa Kajala, and Ronald Pierik:** writing – review and editing. All authors read and have approved the final manuscript.

## Conflicts of Interest

The authors declare no conflicts of interest.

## Peer Review

The peer review history for this article is available in the Supporting Information for this article.

## Supporting information


**Data S1.** Peer review.

**Table S1:** DEGs (limma‐voom output)—both FR‐responsive DEGs AND pith‐specific DEGs.
**Table S2:** GO enrichment analysis—FR‐responsive and pith‐specific.
**Table S3:** Sequences of the synthesized promoters.
**Table S4:** qPCR primers used in this study.

**Figure S1:** The number of differentially expressed genes (DEGs) between the Moneymaker and M82 cultivars in tomato internodes at each sampled timepoint and treatment.

**Figure S2:** GO enrichment analysis of FR‐responsive (WL + FR vs. WL) DEG in the central cylinder of each cultivar.

**Figure S3:** GO enrichment analysis of central cylinder‐specific DEGs, focusing on the comparison between the central cylinder (CC) and the entire internode.

**Figure S4:** Shoot traits measured in multiple dicot species under WL and WL + FR treatments.

**Figure S5:** Cross section of pea ( *P. sativum* ) internode and cell type identification.

**Figure S6:** Fold change of transcript abundance of Solyc07g053450 homologs in response to FR treatment.

**Figure S7:** Fold change of transcript abundance of Solyc08g080150 homologs in response to FR treatment.

**Figure S8:** Fold change of transcript abundance of Solyc01g090760 homologs in response to FR treatment.

## Data Availability

The RNA sequencing data from this study are openly available in NCBI GEO repository reference number GSE255611.
