# Supplementary figures and images for: Effect of Low Red‐to‐Far‐Red Light on Stem Elongation and Pith Cell Development in Dicots
Source: Plant Direct. 2025 Apr 15;9(4):e70072. doi: 10.1002/pld3.70072 (PMC11999800; doi:10.1002/pld3.70072)

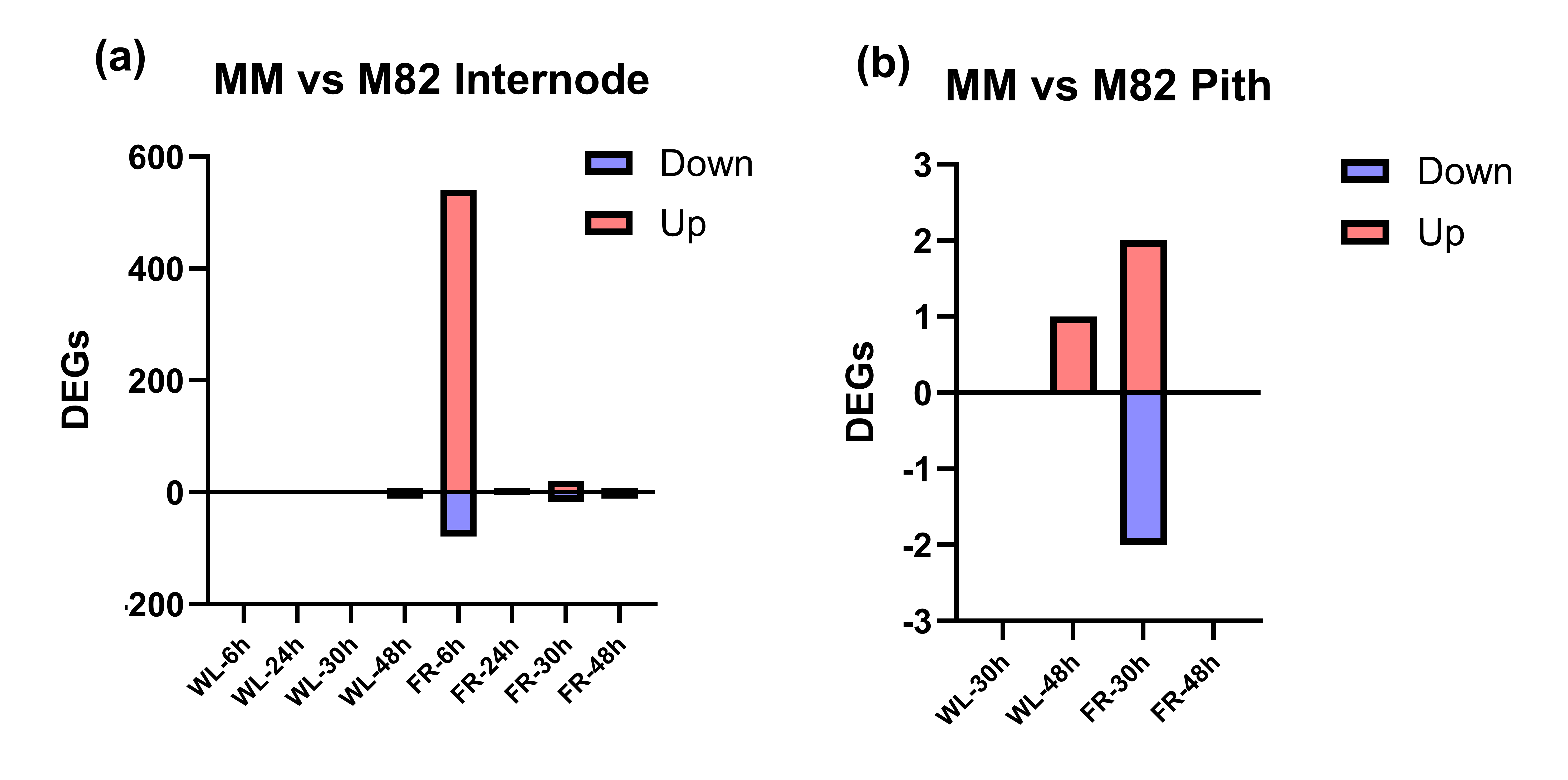

Supplement: Supplementary file 3 — Figure S1: The number of differentially expressed genes (DEGs) between the Moneymaker and M82 cultivars in tomato internodes at each sampled timepoint and treatment. [file PLD3-9-e70072-s002.tif]

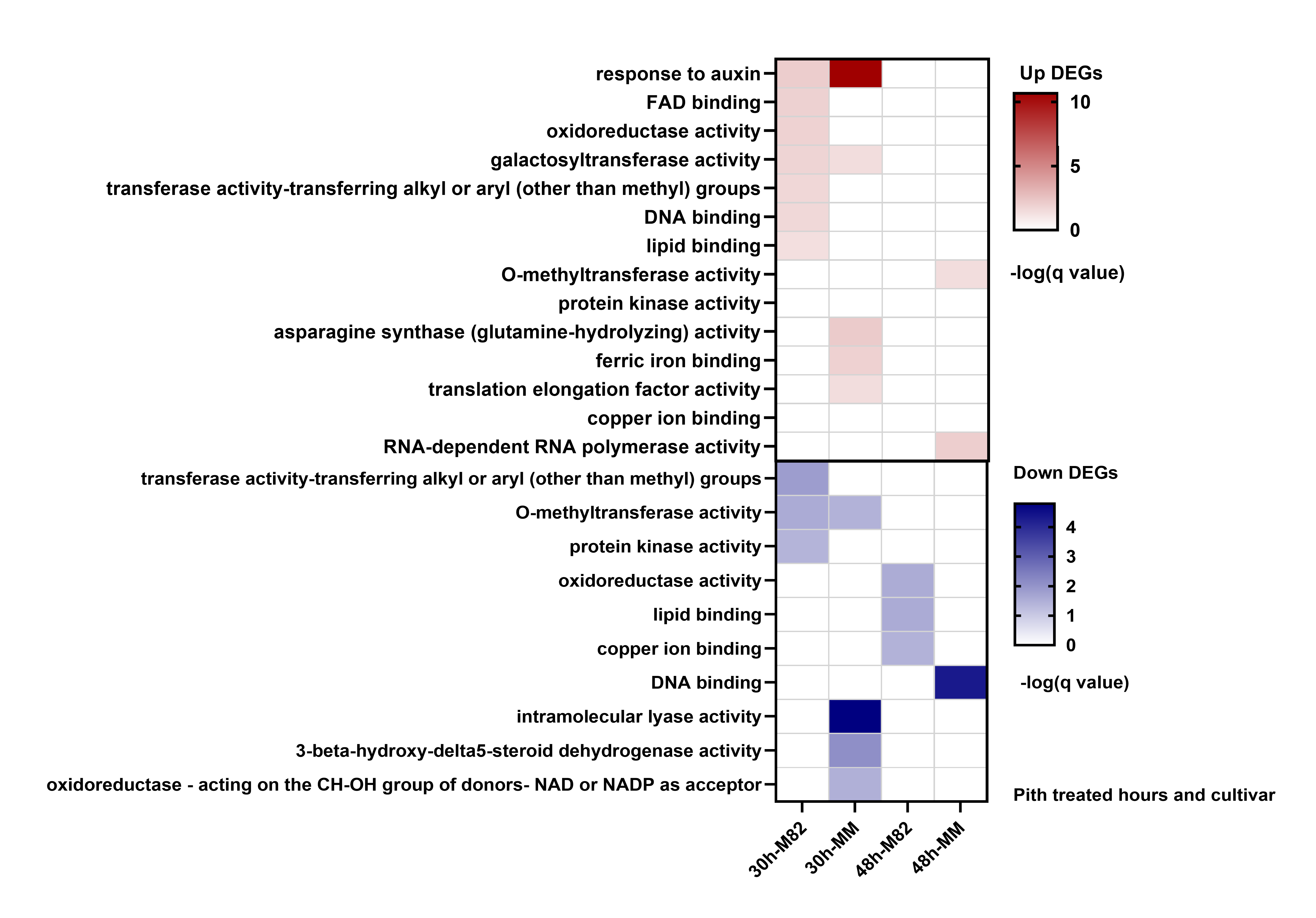

Supplement: Supplementary file 4 — Figure S2: GO enrichment analysis of FR‐responsive (WL + FR vs. WL) DEG in the central cylinder of each cultivar. [file PLD3-9-e70072-s006.tif]

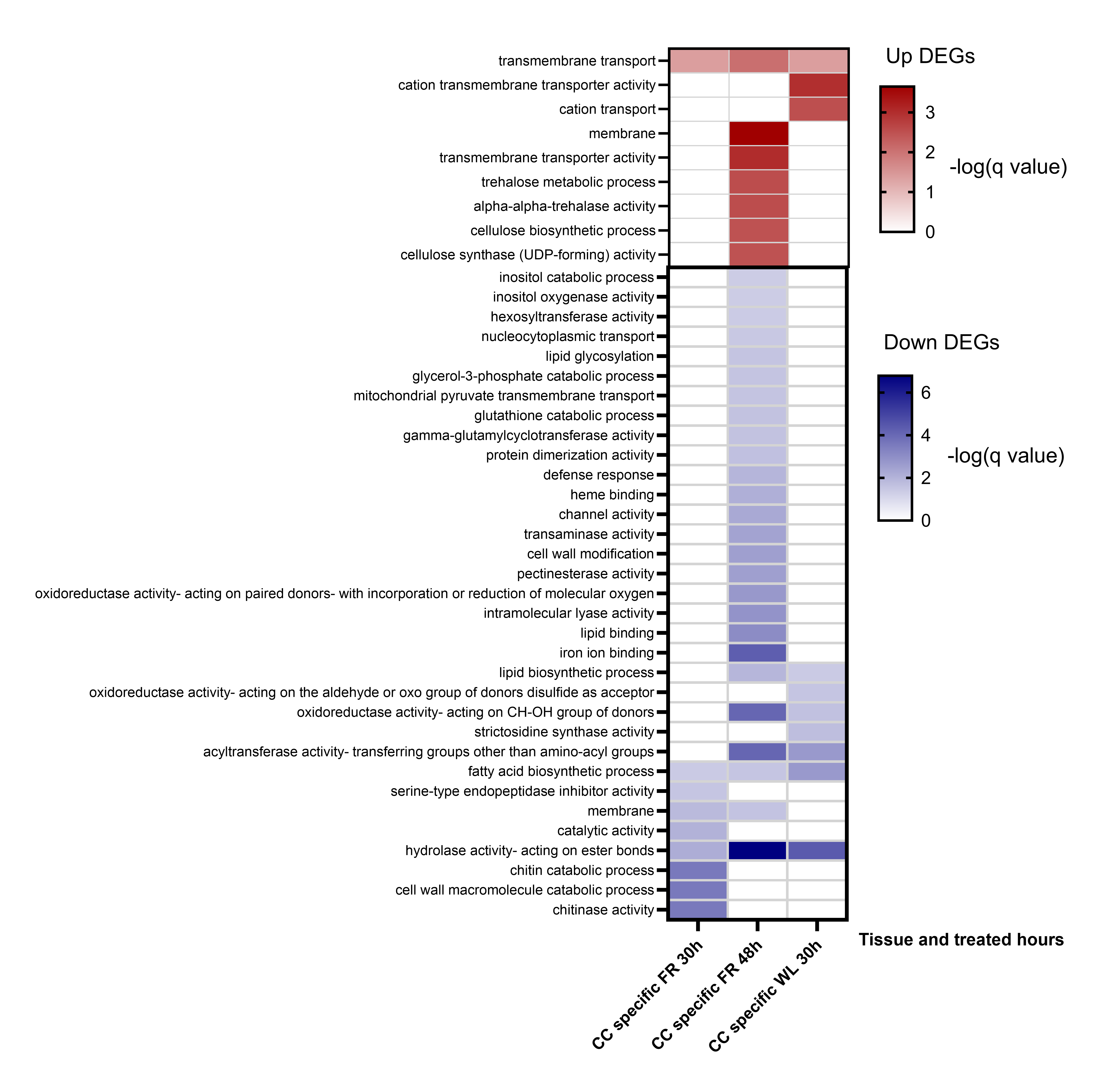

Supplement: Supplementary file 5 — Figure S3: GO enrichment analysis of central cylinder‐specific DEGs, focusing on the comparison between the central cylinder (CC) and the entire internode. [file PLD3-9-e70072-s009.tif]

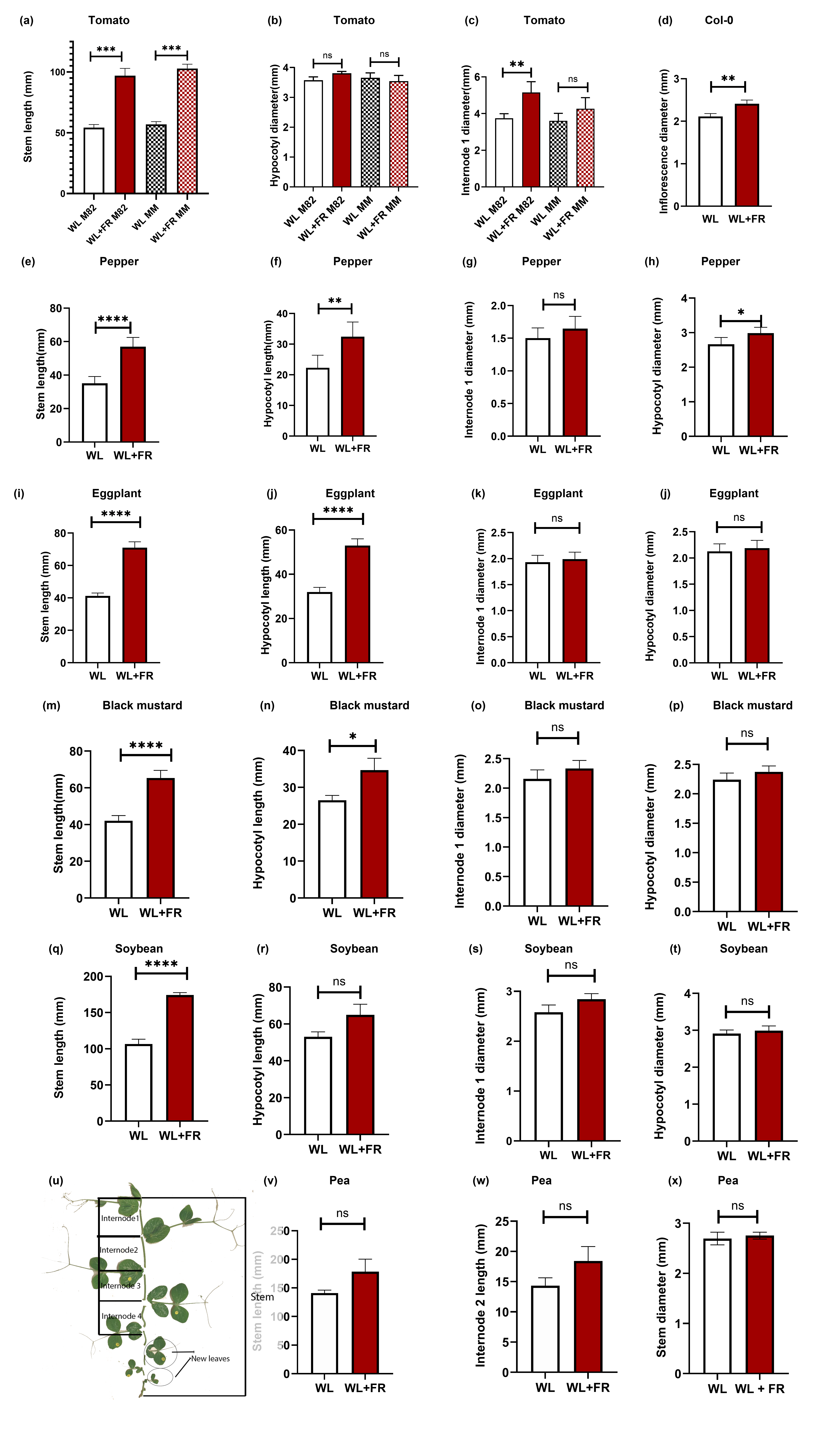

Supplement: Supplementary file 6 — Figure S4: Shoot traits measured in multiple dicot species under WL and WL + FR treatments. [file PLD3-9-e70072-s008.tif]

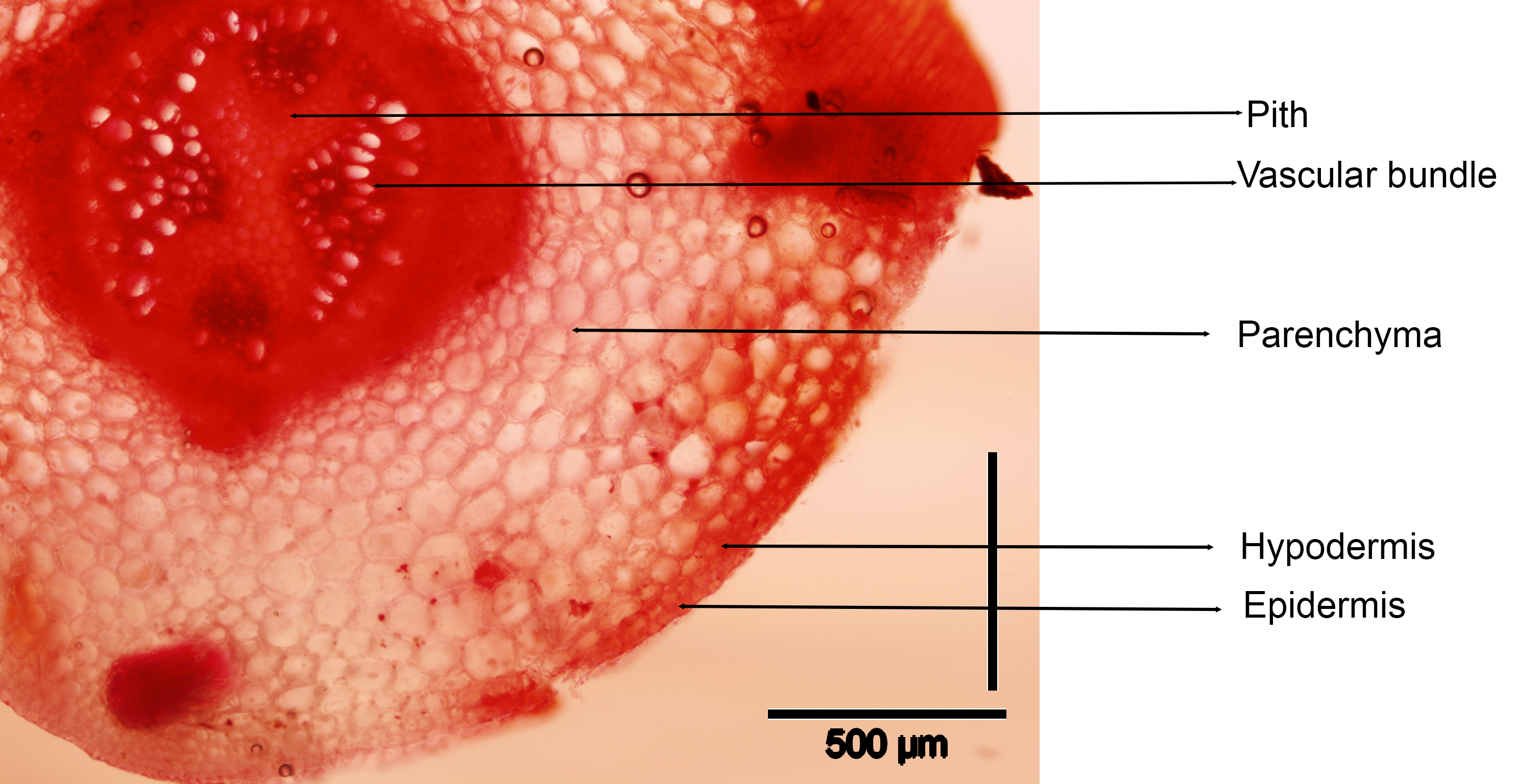

Supplement: Supplementary file 7 — Figure S5: Cross section of pea ( P. sativum ) internode and cell type identification. [file PLD3-9-e70072-s001.tif]

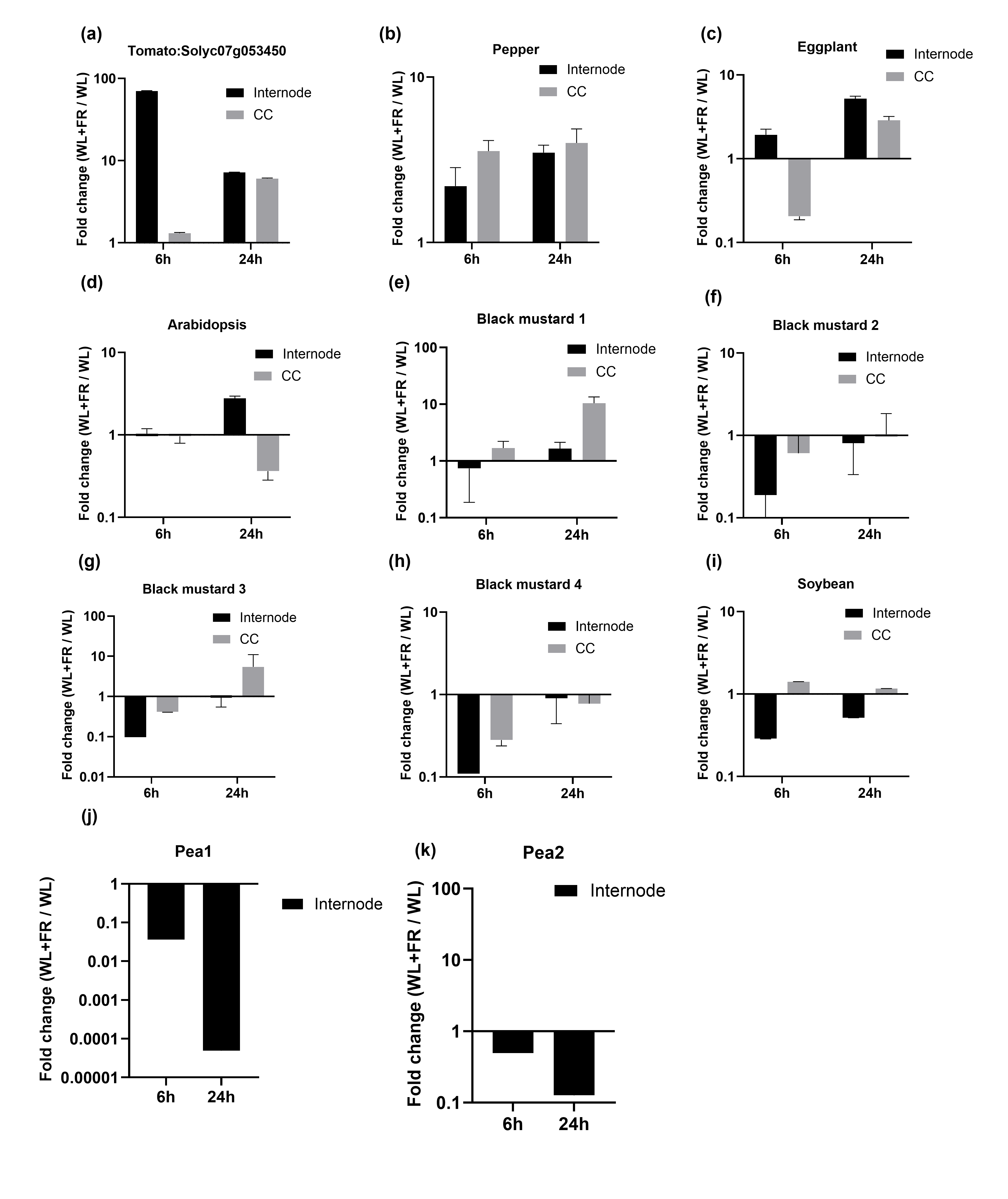

Supplement: Supplementary file 8 — Figure S6: Fold change of transcript abundance of Solyc07g053450 homologs in response to FR treatment. [file PLD3-9-e70072-s005.tif]

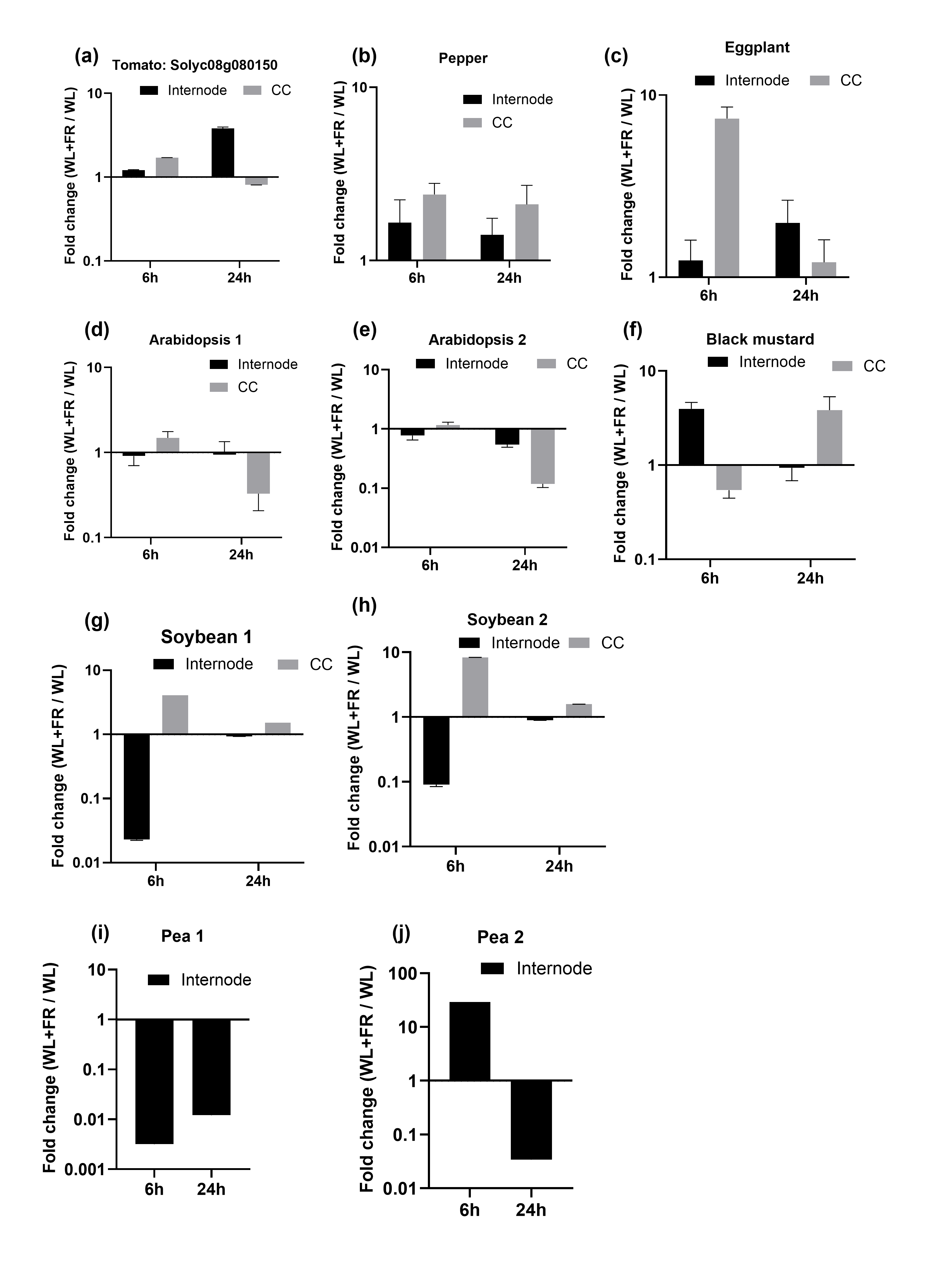

Supplement: Supplementary file 9 — Figure S7: Fold change of transcript abundance of Solyc08g080150 homologs in response to FR treatment. [file PLD3-9-e70072-s003.tif]

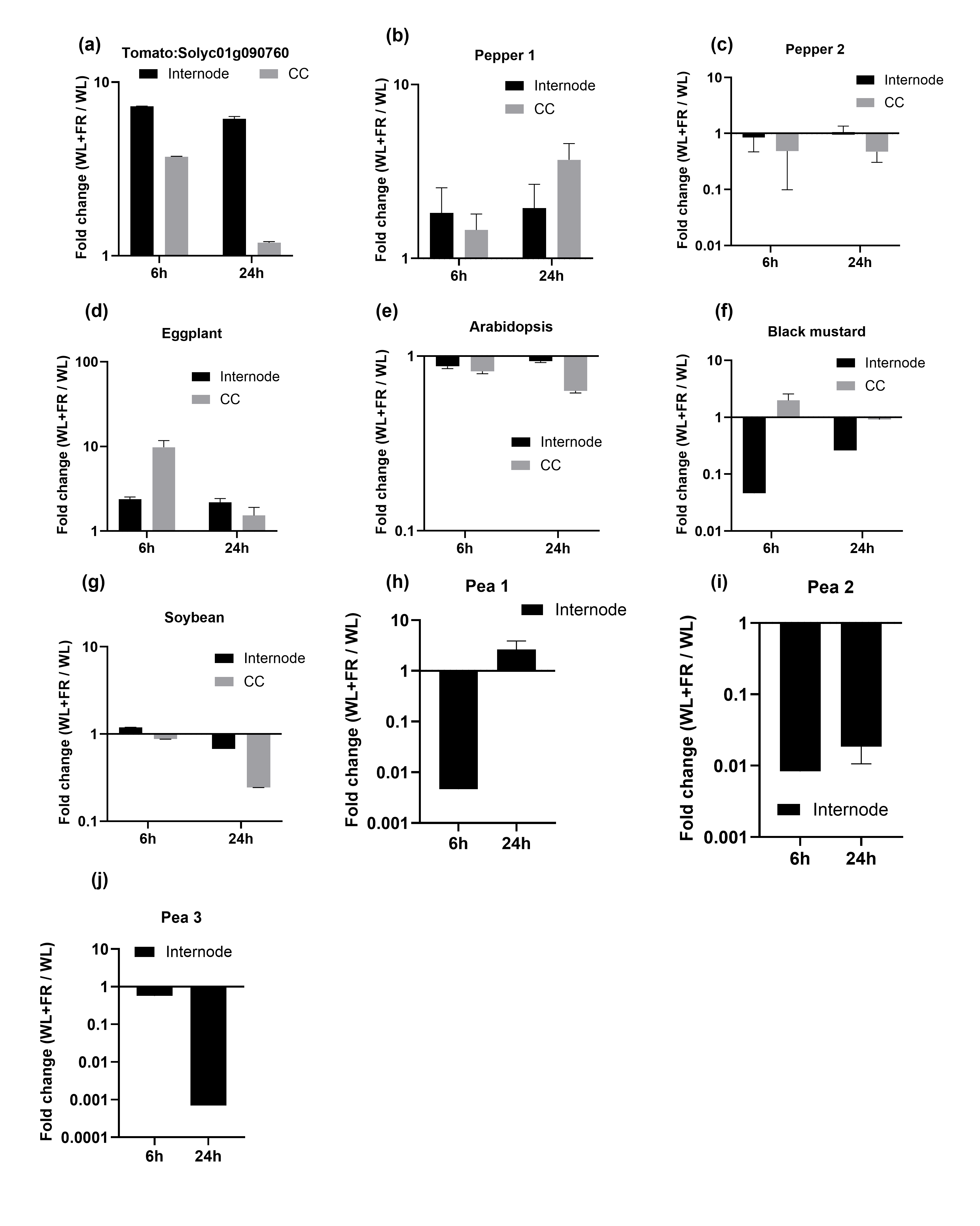

Supplement: Supplementary file 10 — Figure S8: Fold change of transcript abundance of Solyc01g090760 homologs in response to FR treatment. [file PLD3-9-e70072-s010.tif]
