# Supplementary material for: Effect of Low Red‐to‐Far‐Red Light on Stem Elongation and Pith Cell Development in Dicots
Source: Plant Direct. 2025 Apr 15;9(4):e70072. doi: 10.1002/pld3.70072 (PMC11999800; doi:10.1002/pld3.70072)
Supplement: Supplementary file 1 — Data S1. Peer review. [file PLD3-9-e70072-s004.pdf]

Plant Direct
Open Access

**Title**: Effect of low red-to-far-red light on stem elongation and pith cell development in dicots

**Authors:** Linge Li[1,2], Yorrit van de Kaa[1], Lotte van der Krabben [1], Ronald Pierik[1,3], Kaisa Kajala[1,*].

**Decision Letter Round 1:**

February 4, 2025

Dr. Kaisa Kajala
Universiteit Utrecht
Padualaan 8
Utrecht, N/A 3584 CH
Netherlands

RE: Pith cell responses to low red: far-red light in dicot stems

Dear Dr. Kajala:

Thank you for submitting to Plant Direct. All required reviews have been returned and we have now finished our evaluation of your manuscript. In light of the reviewers' and editor's comments, further revisions are needed before the paper can be accepted for publication in Plant Direct.

Please view the editors' and reviewers' comments below and use their suggestions as a guide while you work on your revision.

When uploading the revised version of this article, please be sure to include the following:

-A word document that contains your response to the reviewers. You should respond to each reviewer comment and note the changes made to the manuscript. If you do not agree with a reviewer's comment and choose not to make a suggested revision, please explain why. Please try to provide as complete an answer as possible to each reviewer's criticisms in the "response to reviewers" section.
-A tracked changes document
- A clean version of the latest version of the manuscript

To upload your revision, please click the link below.
https://plantdirect.msubmit.net/cgi-bin/main.plex?el=A7Lr6lk6A1HOe1I4A9ftdOezH2qVxCVAeyfEOO8WI1wZ

In order to provide as timely a service as possible, we ask that your revision is resubmitted within three months after receipt of this request. If an extension is needed, please send a request, along with a brief explanation, to the editorial office at plantdirect@wiley.com .

Thank you very much for giving us an opportunity to review your work. I look forward to receiving the next version.

Sincerely,
Hongtao Ji

-------------------------------------------------------------------------- Editor comments:

I have no further comments. Make sure you address the reviewers' comments properly.

-------------------------------------------------------------------------- Reviewer comments:
Reviewer #1:

The MS presented by Li and collaborators highlights the existence of a cell-type specific response to FR light in solanaceae family. This response occurs in the pith cell type and results in the activation of specific transcription factors. Later they study the conservation of this response within the solanaceae family and compare it with the FR response or other relevant groups (Fabaceae and Brassicaceae). Overall, the manuscript is well written and the experiments support their conclusions. However, I consider that it will benefit of further writing work and discussion. I am indicating below my comments for each section.

TITLE: The current title does not inform of the key conclusions of the study. I recommend generating a more informative title.

INTRODUCTION: This section is very well written, I really enjoyed it. It provides an excellent description of SAS and hypothesis of the evolution of this response. Uses relevant literature. However, I miss mechanistic information of how FR is transduced by phytochromes. This is key for the reader to understand how the expression of the TF can be activated by FR.

MATERIALS AND METHODS: Please correct:
- Line 115: Use Columbia instead of Colombia
- Line 118: The SD treatment does come complete a 24h cycle. I assume authors meant 8h light, 16h dark.

RESULTS:
- Lines 249-252. add more detail/clarity to the results obtained. I recommend sub-grouping the results based on the type of histological section performed (radial vs longitudinal). Then explain why the research is later focused on the pith. This will allow a better transition to the second section of results.
- Authors should provide a representative image for the histological sections performed for each treatment (WL vs WL+FT) on Figure 1.
- RNAseq section needs more detail.
o Line 271: Describe more effectively the experimental set up.
o Lines 278-onwards: How many genes where differentially expressed on each time point? How many were up- and down-regulated?
o Lines 291-292: Explain further
o Lines 294 - 295: indicate the number of TF identified, provide information of the families. Is there any enriched family? Indicate the level of induction for each TF.
- GUS expression analysis of both TF needs to provide comparison between WL and WL + FR
- Figure 4: Add gene names on top of each heatmap.

DISUCUSSION:
I find quite interesting that pea does not respond to FR treatments (Fig 3J). Can authors elaborate more about this observation? Have they searched for differences in photoreceptor composition?

Reviewer #2:

The article is devoted to the study of the response of stem pith cells in dicotyledonous plants to low red to far-red light ratio (R:FR) in the context of shade avoidance syndrome (SAS). The authors examined in detail the anatomical and transcriptomic changes occurring in the tomato pith under the influence of low R:FR and identified specific transcription factors conserved within the Solanaceae family.
The main merit of the article is that the authors for the first time focused on the unique role of pith cells in SAS and described family-specific mechanisms associated with the expression of transcription factors GATA, TCP and bZIP. These data significantly deepen our understanding of the molecular basis of plant adaptation to competitive light conditions.
However, the article has many shortcomings that require revision. The purpose of the study is not clearly formulated. Despite the interest in pith cells, their choice as an object of study is not sufficiently justified, especially given the known importance of the epidermis and vascular tissues in SAS. In addition, the introduction contains redundant discussions of evolutionary aspects that are not directly related to the main topic.
The methodological section needs significant improvement. There is no information on how the R:FR

ratio was calculated. It is not specified whether the spectral peaks (e.g. 660 and 730 nm) or the integrated intensity in the corresponding spectral regions were used. This is a key aspect, especially when comparing the conditions for tomatoes, Arabidopsis and Brassica nigra, where different combinations of red and far-red light were used. It is also unclear why such a low light intensity (200 $\mu mol\ m^{-2}\ s^{-1}$) was used for tomatoes, given that optimal conditions for their growth require more than 500 $\mu mol\ m^{-2}\ s^{-1}$.
The duration of the experiment needs to be clarified: how long the plants were exposed to R:FR and under what conditions they were grown before the experiment. If the plants were initially grown under standard lighting conditions, this could have significantly affected their initial physiological parameters.
The results of the study are presented insufficiently convincingly. Descriptions of changes in the anatomy of core cells and their gene expression are not clear: there are no qualitative and quantitative graphical materials. The analysis of molecular data, although it identifies key transcription factors, is limited by the lack of functional validation. Experiments with knockouts or overexpression lines were not carried out, which makes conclusions about the role of GATA, TCP and bZIP preliminary.
In addition, the inclusion of a large number of plant species from different families in the study requires an analysis of common and unique mechanisms beyond the Solanaceae family. However, the authors did not pay due attention to interfamily comparative aspects, which leaves a significant part of the data uninterpreted.
The Materials and Methods section also lacks information on the spectral characteristics of the light sources, which makes it difficult to reproduce the experiments. The tables with differentially expressed genes (DEGs) do not contain data on statistical significance, which reduces the confidence in the presented results.

**Decision Letter Round 2:**

April 6, 2025

Dr. Kaisa Kajala
Universiteit Utrecht
Padualaan 8
Utrecht, N/A 3584 CH
Netherlands

MSID: 2024-01668R1
MS TITLE: Effect of low red-to-far-red light on stem elongation and pith cell development in dicots

Dear Dr. Kaisa Kajala:

Plant Direct
Open Access

I am pleased to inform you that your manuscript "Effect of low red-to-far-red light on stem elongation and pith cell development in dicots" has been accepted for publication in Plant Direct.

Your article will appear online in the next available issue of Plant Direct. To ensure your article gets published as quickly as possible, please pay attention to the steps detailed below. We have found that most of the delays happen at this stage, especially at the payment stage, so please respond as quickly as possible when prompted.

License Agreement: Once your article has been accepted it will move to Production and undergo admin and file checking - you may receive an email with any queries we have at this stage. When all required items are received by the publisher and queries resolved, the corresponding author will receive an email from Wiley's Author Services system which will ask them to log on at https://authorservices.wiley.com/bauthor and will present them with the appropriate license for completion. Your article cannot be published until both the signed license agreement and payment of the article fee have been received.

Payment of the Open Access Article Publication Fee: All articles published in Plant Direct are fully open access: immediately and freely available to read, download and share. Plant Direct charges a publication fee to cover the publication costs. The corresponding author for this manuscript should have already received a quote from the payments team (cs-openaccess@wiley.com) with the estimated article publication fee; please email cs-openaccess@wiley.com if this has not been received. The corresponding author should log on to the Wiley Author Services site, where the publication fee can be paid by credit card or an invoice. Pro Forma can also be requested. Payment of the publication charge must be received before the article will be published online.

Proofs: You will have the opportunity to look over your paper once more when you receive the author proofs for your article. The proofs will be with you in approximately two weeks. Please note that, in addition to publishing reviewer comments, the author's responses to review comments will also be published alongside the final version of the paper. If you would not like the author's responses to be published, please contact the editorial office at plantdirect@wiley.com .

Promotion of your article: You can help your research get the attention it deserves! Check out Wiley's free Promotion Guide for best-practice recommendations for promoting your work at www.wileyauthors.com/eeo/guide. And learn more about Wiley Editing Services which offers professional video, design, and writing services to create shareable video abstracts, infographics, conference posters, lay summaries, and research news stories for your research at www.wileyauthors.com/eeo/promotion.

Thank you again for your contribution to Plant Direct. If you have any questions, feel free to contact the editorial office at plantdirect@wiley.com .

Sincerely,

Hongtao Ji

Gustavo MacIntosh

Editor, Plant Direct

---------------------------------------------------------------------------- Editor comments

---------------------------------------------------------------------------- Reviewer comments:

Reviewer #1:

Authors have address my comments in the revised version of the MS.

Reviewer #2:

The article has been completely revised, and I have no further comments.

**Response to Reviewer:**

Dear editorial team & reviewers,

We thank you for the careful reading of our manuscript, and we hope that we have addressed your concerns in the edited manuscript. Below, we address the reviewer comments individually, indicating in yellow highlights the updates and edits made into the manuscript.

Reviewers' comments to the author:

**Reviewer: 1**

Plant Direct

Open Access

Comments to the Author

The MS presented by Li and collaborators highlights the existence of a cell-type specific response to FR light in solanaceae family. This response occurs in the pith cell type and results in the activation of specific transcription factors. Later they study the conservation of this response within the solanaceae family and compare it with the FR response or other relevant groups (Fabaceae and Brassicaceae). Overall, the manuscript is well written and the experiments support their conclusions. However, I consider that it will benefit of further writing work and discussion. I am indicating below my comments for each section.

We thank the reviewer for their kind words and their thoughtful comments on the manuscript.

1. TITLE: The current title does not inform of the key conclusions of the study. I recommend generating a more informative title.

We agree that the original title did not sufficiently convey the key conclusions of our study. To address this, we have revised the title to better reflect the main findings and focus of our research. The new title is: ***"Effect of low red-to-far-red light on stem elongation and pith cell development in dicots"*** We believe this revised title more accurately highlights the central theme of our work.

2. INTRODUCTION: This section is very well written, I really enjoyed it. It provides an excellent description of SAS and hypothesis of the evolution of this response. Uses relevant literature. However, I miss mechanistic information of how FR is transduced by phytochromes. This is key for the reader to understand how the expression of the TF can be activated by FR.

We have added the following section to the introduction to address this relevant remark (starting on line 55):

*Phytochromes are 120-kD soluble proteins that have a covalently linked linear tetrapyrrole chromophore. They exist in two photointerconvertible forms, Pr and Pfr. Pr is the R light-absorbing form, which can be converted to Pfr (the FR-absorbing form) by the R light. Pfr is the active form of phytochrome, and can be converted back to Pr by FR light (Reed et al., 1993; Kiss et al., 2003). The active form Pfr translocases from the cytosol into the nucleus and interacts with PHYTOCHROME INTERACTING FACTORS (PIFs) and facilitates their inactivation and degradation. PIFs are growth-promoting transcription factors involved in various physiological processes that accumulate in response to increase in FR light and lead to SAS phenotypes (Shin et al., 1997; Ruberti et al., 2012; Yang and Li, 2017).*

3. RESULTS:

- Lines 249-252. add more detail/clarity to the results obtained. I recommend sub-grouping the results based on the type of histological section performed (radial vs longitudinal). Then explain why the research is later focused on the pith. This will allow a better transition to the second section of results.

We have reordered the heatmap based on the type of histological section performed (radial vs. longitudinal). This reorganization provides a clearer presentation of the findings and highlights the distinct insights gained from each type of section. Additionally, we have added explanation why we focus on the pith (now on lines 272-275):

*As reported in Arabidopsis, FR-induced elongation occurs in the petiole epidermis, and we observed a similar response in tomato. However, pith cells displayed both elongation and pronounced increase in cell layer number—a less explored phenomenon—prompting us to focus our investigation on this cell type.*

4. RNAseq section needs more detail.

Line 271: Describe more effectively the experimental set up.

We have addressed the reviewer's comments with following addition (lines 277-280):

*We designed an RNA-seq experiment with four timepoints (6h, 24h, 30h, and 48h after the start of the light treatment) and harvested the first internode or its central cylinder from tomato stems for RNA extraction. This approach allowed us to identify the gene expression changes accompanying the internode elongation and the pith responses.*

5 Lines 278-onwards: How many genes where differentially expressed on each time point? How many were up- and down-regulated?

Given that the DEG numbers can be observed in Figures 2b and 2C, and the full list of the genes is accessible in the supplementary data, we have added only some of the DEG numbers into the text as follows (lines 286-288):

*Notably, at the 6-hour mark, the most DEGs were observed in the whole internode (153 up, 138 down), followed by a general trend of decreasing DEGs over time (to 62 up, 51 down at 48h). The central cylinder followed a similar pattern, with more DEGs at 30 h (76 up, 77 down) than at 48 h (50 up, 49 down). Next, we tested the gene expression of the central cylinder against the whole internode for each treatment and timepoint (Figure 2c) and found 26 central cylinder-enriched genes.*

6 Lines 291-292: Explain further

We have addressed the reviewer's comments with following explanation:

*We also tested GO term enrichment of the central cylinder-enriched DEGs (Figure S3) for each time point and treatment, and GO category "transmembrane transport" was enriched throughout the central cylinder-enriched DEGs, either reflecting the little understood roles of pith in transporting molecules, or the effect from the residual vascular tissues with known transport roles included in the sampling.*

7 Lines 294 - 295: indicate the number of TF identified, provide information of the families. Is there any enriched family? Indicate the level of induction for each TF.

We have addressed the TF number and TF families (now on lines 303-307). The level of induction of the selected TFs are shown in Fig 2e-g, and including this information in the table for every 46 TFs would reduce the readibility of Table 1. The full data are available (i.e. the fold changes and adjusted p-values for each DE comparison) are available in the Supplementary table S1.

*"We further examined the DEG lists and identified 46 FR-responsive transcription factor (TF) encoding genes in our data (Table 1). TFs typically coordinate and amplify responses to environmental cues, so we focused our follow-up inquiry on selected TFs of interest. The dataset contains 10 bHLH (Basic helix-loop-helix) transcription factors, 8 MYB transcription factors, 4 basic leucine zipper transcription factors, and 3 Homeobox-leucine zipper-like transcription factors."*

8. GUS expression analysis of both TF needs to provide comparison between WL and WL + FR

Unfortunately, our limited experimental materials (number of transgenic seeds obtained) caused problems with this experiment. We conducted GUS expression analysis for both promoter:GUS lines under both WL and WL+FR conditions. However, after the pre-GUS treatment, the samples became highly delicate, making sectioning challenging and compromising the quality of comparisons. Due to these technical limitations, the final images are not publication quality and we opted to present the best-sectioned samples from the WL treatment only. Here are example images for both WL and WL+FR to show what we observed.

Plant Direct
Open Access

*Solyc07g053450*

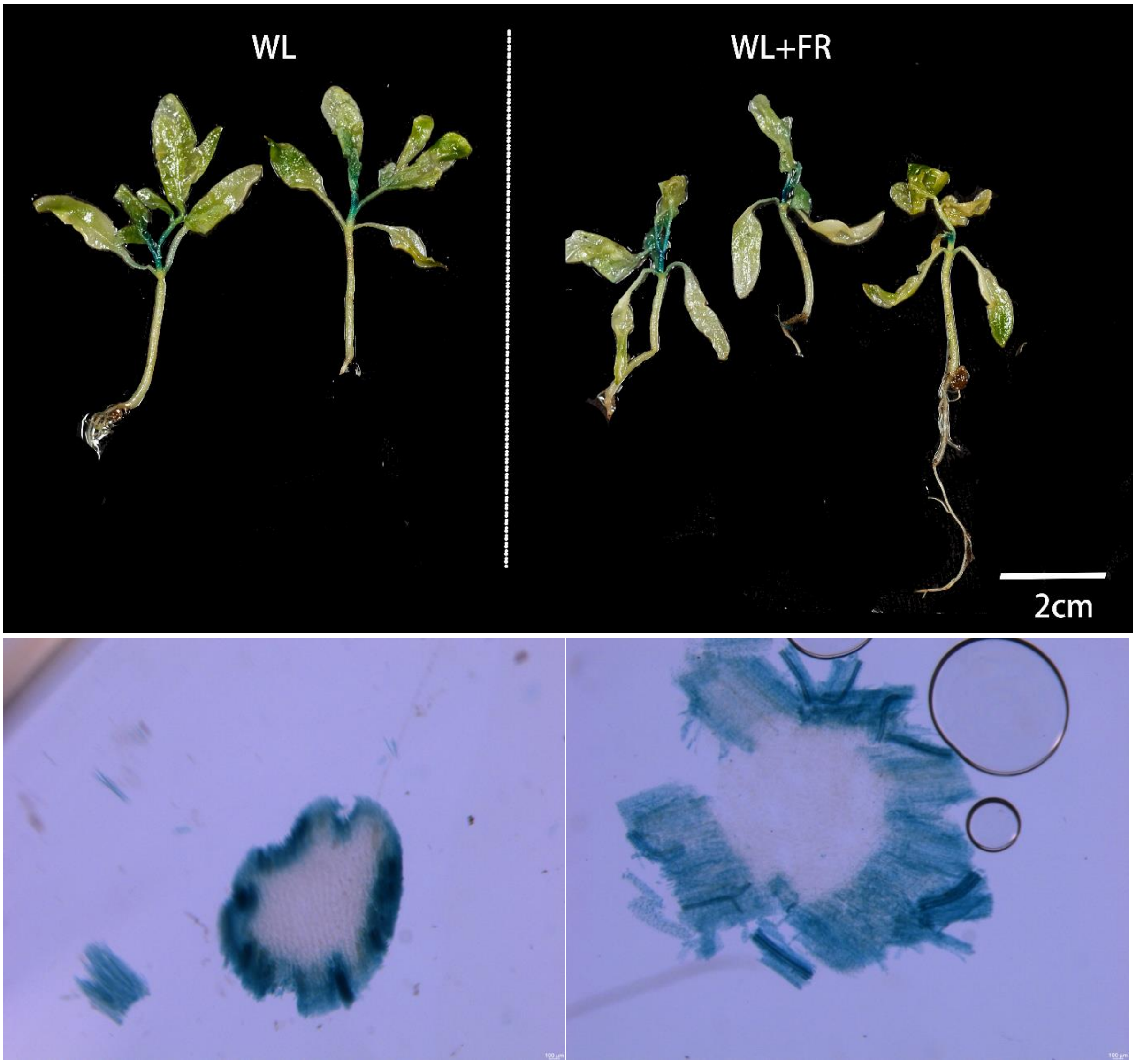


*Solyc01g090760*

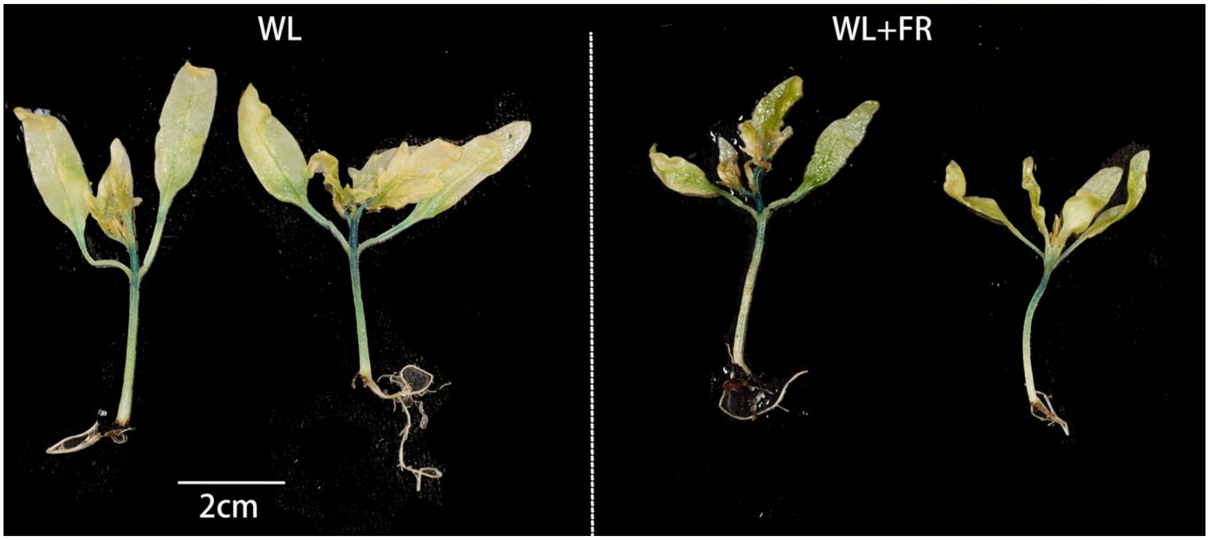


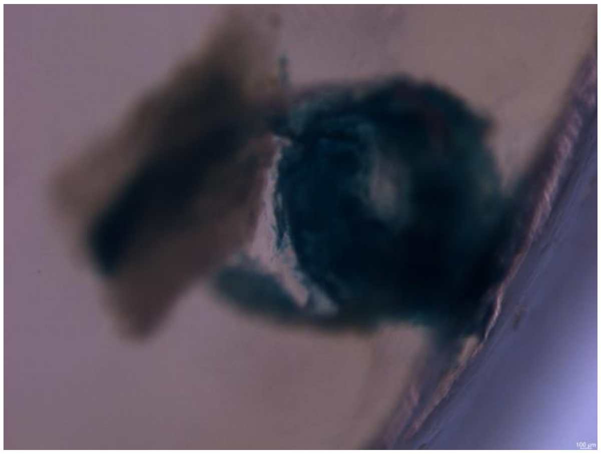

9. DISUCUSSION:

I find quite interesting that pea does not respond to FR treatments (Fig 3J). Can authors elaborate more about this observation? Have they searched for differences in photoreceptor composition?

Thank you for catching this, we have added this discussion section in the revised manuscript \(line 447 onwards).

*Pea photoreceptors, while sharing many similarities with those of other plants, exhibit unique traits such as the duplication of the CRY2 gene—which shows differential regulation under various light conditions—and novel mutants like lfp1-1 (late-flowering photomorphogenic mutant) that display distinct phenotypes under blue light, highlighting differences in how peas integrate light signals compared to other species (Platten, 2003). However, further research is needed to fully characterize these differences and their functional implications.*

Minor:

We thank the reviewer for their careful reading of the manuscript and noticing the typos and mistakes. The below comments have all been addressed in the updated manuscript.

- Line 115: Use Columbia instead of Colombia updated
- Line 118: The SD treatment does come complete a 24h cycle. I assume authors meant 8h light, 16h dark. correct - updated
- Authors should provide a representative image for the histological sections performed for each treatment (WL vs WL+FT) on Figure 1. updated
- Figure 4: Add gene names on top of each heatmap. updated

**Reviewer: 2**

Comments to the Author

The article is devoted to the study of the response of stem pith cells in dicotyledonous plants to low red to far-red light ratio (R:FR) in the context of shade avoidance syndrome (SAS). The authors examined in detail the anatomical and transcriptomic changes occurring in the tomato pith under the influence of low R:FR and identified specific transcription factors conserved within the Solanaceae family.

The main merit of the article is that the authors for the first time focused on the unique role of pith cells in SAS and described family-specific mechanisms associated with the expression of transcription factors GATA, TCP and bZIP. These data significantly deepen our understanding of the molecular basis of plant adaptation to competitive light conditions.

We thank the reviewer for their kind words and their thoughtful comments on the manuscript.

Comments:

1) However, the article has many shortcomings that require revision. The purpose of the study is not clearly formulated. Despite the interest in pith cells, their choice as an object of study is not sufficiently justified, especially given the known importance of the epidermis and vascular tissues in SAS. In addition, the

introduction contains redundant discussions of evolutionary aspects that are not directly related to the main topic.

Firstly, we originally did not set out to study pith cell specifically but any cell type that showed the strongest response. We now realize that the sentences describing the pith findings and the focus on them were not included when editing a thesis chapter into this manuscript, and this justification to work on pith is now included on lines 272-275:

*As reported in Arabidopsis, FR-induced elongation occurs in the petiole epidermis, and we observed a similar response in tomato. However, pith cells displayed both elongation and pronounced increase in cell layer number—a less explored phenomenon—prompting us to focus our investigation on this cell type.*

We also edited the introduction to clarify this open-ended approach (lines 91-94):

*We paired organ-level measurements with cellular anatomy, looking for the cell types that responded to low R:FR. We discovered changes in the pith cell length and layer number in response to low R:FR. We then profiled both the internode and the central cylinder (mainly composed of pith) transcriptomes to characterize the pith-related transcriptional changes.*

Secondly, we realise that the evolutionary discussion may be redundant to the objectives of this research, but at the same time, Reviewer 1 enjoyed reading this part as a lead-in to the article. We leave it to the editor to decide if this paragraph (lines 67-86) fits the style of the journal.

2) The methodological section needs significant improvement. There is no information on how the R:FR ratio was calculated. It is not specified whether the spectral peaks (e.g. 660 and 730 nm) or the integrated intensity in the corresponding spectral regions were used. This is a key aspect, especially when comparing the conditions for tomatoes, Arabidopsis and Brassica nigra, where different combinations of red and far-red light were used. It is also unclear why such a low light intensity (200 µmol $m^{-2}$ $s^{-1}$) was used for tomatoes, given that optimal conditions for their growth require more than 500 µmol $m^{-2}$ $s^{-1}$.

To maintain consistency with the experimental conditions used for Arabidopsis, which is a model organism in plant biology, we adopted a similar light intensity. Due to equipment constraints in our laboratory, 200 µmol $m^{-2}$ $s^{-1}$ was the maximum achievable intensity in our growth cabinets while maintaining stable and uniform light distribution across all treatments.

While optimal growth conditions for tomatoes typically require higher light intensities (>500 µmol $m^{-2}$ $s^{-1}$), this study focused on the relative effects of R:FR ratio rather than absolute growth rates. The chosen

intensity was sufficient to elicit measurable physiological responses to far-red light without causing light stress.

Details of how we calculated R:FR ratio is added to methodology as follows (lines 128-133):

*The red to far-red (R:FR ) ratio was calculated to quantify the relative abundance of red light (660 nm) to far-red light (730 nm) in the light spectrum. Spectral irradiance data were collected using a calibrated spectroradiometer or equivalent instrument. The red irradiance value was divided by the far-red irradiance value to obtain the ratio. This method is consistent across all experiments involving all species, allowing for direct comparisons between species.*

3) The duration of the experiment needs to be clarified: how long the plants were exposed to R:FR and under what conditions they were grown before the experiment. If the plants were initially grown under standard lighting conditions, this could have significantly affected their initial physiological parameters.

We appreciate this comment and have clarified the experimental duration and growth conditions in the revised manuscript as follows (lines 123-127):

*All plants were initially grown under standard white light conditions (with a balanced R:FR ratio) until they reached a uniform developmental stage. This ensured that all plants had similar physiological starting points before the far-red light treatment (which was applied during the light period (16h/day), and lasted for a week for the phenotypic measurements).*

4) The results of the study are presented insufficiently convincingly. Descriptions of changes in the anatomy of core cells and their gene expression are not clear: there are no qualitative and quantitative graphical materials. The analysis of molecular data, although it identifies key transcription factors, is limited by the lack of functional validation. Experiments with knockouts or overexpression lines were not carried out, which makes conclusions about the role of GATA, TCP and bZIP preliminary.

We fully agree – to show the role of the target TFs for SAS would require knockout or overexpression experiments, but this is something we do not have the experimental capacity for, especially with tomato as the model. Hence, we do not make any claims of their role – beyond the FR-responsive expression patterns and their conservation in Solanaceae – in the manuscript.

5) In addition, the inclusion of a large number of plant species from different families in the study requires an analysis of common and unique mechanisms beyond the Solanaceae family. However, the authors did not pay due attention to interfamily comparative aspects, which leaves a significant part of the data uninterpreted.

We appreciate the reviewer's suggestion to further explore interfamily comparative aspects. However, given that we carried out RNAseq only in tomato, it is difficult to identify the genes/TFs unique to other families. Hence, we are able to only test the conserved responses of our tomato target TFs, and not identify the what may be unique to the other families. Morphologically, in our sample species Solanaceae family exhibited morphological uniformity, while the species in the Fabaceae and Brassicaceae families do not share consistent stem morphology. A comprehensive analysis of common and unique mechanisms across families would require a larger and more diverse sample set, which is beyond the scope of our work. We have now clarified this limitation in the revised manuscript (lines 473-476).

*Then, to identify what may be the common responses between these families and the unique FR-responsive TFs in Fabaceae and Brassicaceae, a wider RNAseq profiling targeting species from those families would be an interesting follow-up.*

6) The Materials and Methods section also lacks information on the spectral characteristics of the light sources, which makes it difficult to reproduce the experiments. The tables with differentially expressed genes (DEGs) do not contain data on statistical significance, which reduces the confidence in the presented results.

Thank you for your valuable feedback. As stated in the manuscript, the spectral characteristics of the light sources is described in our recent publication, Li et al., 2024 (Figure 1A and section 2.1) – which we opted not to copy into this manuscript to avoid self-plagiarism. Similarly for the DE analysis, the details are given in Li et al 2024, but indeed it is important to state the cut-off of adjusted p-value of <0.05 (now on line 160). In addition, the statistical significance of all differentially expressed genes (DEGs) is provided in Table S1.

Sincerely, also on behalf of all the authors,

Kaisa Kajala
